# Dose Response Relationship in Anti-Stress Gene Regulatory Networks

**DOI:** 10.1371/journal.pcbi.0030024

**Published:** 2007-03-02

**Authors:** Qiang Zhang, Melvin E Andersen

**Affiliations:** Division of Computational Biology, CIIT Centers for Health Research, Research Triangle Park, North Carolina, United States of America; University of Toronto, Canada

## Abstract

To maintain a stable intracellular environment, cells utilize complex and specialized defense systems against a variety of external perturbations, such as electrophilic stress, heat shock, and hypoxia, etc. Irrespective of the type of stress, many adaptive mechanisms contributing to cellular homeostasis appear to operate through gene regulatory networks that are organized into negative feedback loops. In general, the degree of deviation of the controlled variables, such as electrophiles, misfolded proteins, and O_2_, is first detected by specialized sensor molecules, then the signal is transduced to specific transcription factors. Transcription factors can regulate the expression of a suite of anti-stress genes, many of which encode enzymes functioning to counteract the perturbed variables. The objective of this study was to explore, using control theory and computational approaches, the theoretical basis that underlies the steady-state dose response relationship between cellular stressors and intracellular biochemical species (controlled variables, transcription factors, and gene products) in these gene regulatory networks. Our work indicated that the shape of dose response curves (linear, superlinear, or sublinear) depends on changes in the specific values of local response coefficients (gains) distributed in the feedback loop. Multimerization of anti-stress enzymes and transcription factors into homodimers, homotrimers, or even higher-order multimers, play a significant role in maintaining robust homeostasis. Moreover, our simulation noted that dose response curves for the controlled variables can transition sequentially through four distinct phases as stressor level increases: initial superlinear with lesser control, superlinear more highly controlled, linear uncontrolled, and sublinear catastrophic. Each phase relies on specific gain-changing events that come into play as stressor level increases. The low-dose region is intrinsically nonlinear, and depending on the level of local gains, presence of gain-changing events, and degree of feedforward gene activation, this region can appear as superlinear, sublinear, or even J-shaped. The general dose response transition proposed here was further examined in a complex anti-electrophilic stress pathway, which involves multiple genes, enzymes, and metabolic reactions. This work would help biologists and especially toxicologists to better assess and predict the cellular impact brought about by biological stressors.

## Author Summary

To maintain a stable intracellular environment, cells are equipped with multiple specialized defense programs that are launched in response to various external chemical and physical stressors. These anti-stress mechanisms comprise primarily gene regulatory networks, and like many manmade control devices, such as thermostats and automobile cruise controls, they are often organized into negative feedback circuits. A quantitative understanding of how these control circuits operate in the cell can help us to assess and predict more accurately the cellular impacts brought about by perturbing stressors, such as environmental toxicants. Using control theory and computer simulations, we explored nature's design principle for anti-stress gene regulatory networks, and the manner in which cells respond and adapt to perturbations. We showed that cells can exploit multiple mechanisms, such as protein homodimerization, cooperative binding, and auto-regulation, to enhance the feedback loop gain, which, according to control theory, is a basic principle for effective perturbation resistance. We also illustrated that the steady-state dose response curve is likely to transition through multiple phases as stressor level increases, and that the low-dose region is inherently nonlinear. Our results challenge the common practice of linear extrapolation for evaluating the low-dose effect, and would lead to improved human health risk assessment for exposures to environmental toxicants.

## Introduction

**Figure 1:**
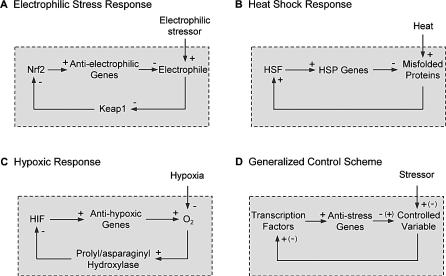
Anti-Stress Gene Regulatory Networks (A–C) Schematic representations of the gene regulatory networks that mediate electrophilic stress response, heat shock response, and hypoxic response, respectively. (D) Generalized negative feedback control scheme for the anti-stress gene regulatory networks in (A–C). HIF, hypoxia-inducible factor.

Cells in vivo must maintain a relatively stable intracellular milieu in an extracellular environment that is constantly changing and is potentially unpredictable. Notably, many intracellular biomolecules need to be held within closely regulated ranges of concentrations for normal cell functions. Examples of these biochemical species, which could be detrimental and/or beneficial to cellular health, are electrophiles, reactive oxygen species (ROS), DNA adducts, misfolded proteins, O_2_, and glucose. When external stressors cause these molecules to deviate from their basal operating concentrations for an extended period of time, normal cell functions become disrupted, and cell cycle arrest and apoptosis may ensue [1]. Homeostatic regulation of vital intracellular biochemical species appears to operate primarily via gene regulatory networks that respond specifically to particular types of physical/chemical insults, such as electrophilic chemicals, heat shock, hypoxia, and hyperosmolarity [2–5]. As with many manmade control devices, such as thermostats and automobile cruise controls, these homeostatic gene regulatory networks are usually organized into negative feedback circuits that can be generalized into a common control scheme (Figure 1). The output of the system, referred to as controlled variable, is the biochemical species that is perturbed by external stressors and therefore needs to be tightly controlled. The system contains specific transcription factors that serve as transducers to either directly or indirectly sense the level of the controlled variable. In this fashion, alterations in the concentration of the controlled variable affect the activity or abundance of the transcription factor. Activated transcription factors then upregulate expression of individual or suites of anti-stress genes, many of which encode enzymes that participate in an array of interconnected biochemical reactions to counteract the perturbation of the controlled variable.

Control and dynamic system theory has benefited applied fields such as electronic and mechanical engineering for many decades, and in recent years increasing efforts have been made to apply similar concepts to biological systems including adaptive responses [6–14]. Our goal is to understand nature's design principle for anti-stress cellular homeostasis and to improve prediction of the disrupting effects of biological stressors. Of practical importance for risk assessment at the cellular level is the steady-state dose response relationship between stressor levels and various measurable biochemical endpoints including the controlled variables, transcription factors, and gene expression. Cell responses in the low-dose region are particularly relevant to human health risk assessment, and it is traditionally difficult to explain and predict dose response behaviors in this region due to uncertainty and subtlety of the curvature. To accurately describe and fully understand complex dose response behaviors, the underlying biochemical networks will have to be examined through quantitative models. With respect to the mathematical approaches involved, theoretical development in quantitative analysis of controls in biochemical networks, including metabolic control analysis (MCA) and biochemical systems theory (BST), has proven to be of great value [15–18]. Using numerical simulation and concepts from MCA, BST, and classical control theory, the present study focused on understanding the quantitative basis for the steady-state dose response in an anti-stress gene regulatory network. While some of the conclusions presented in this paper may seem implicitly familiar, or even obvious, to engineers, they nonetheless provide an important framework by which biologists and especially toxicologists can improve the accuracy with which they evaluate the influence of biological stressors on intracellular control processes under different exposure conditions.

## Result

### Primer: Response Coefficient (Gain) and Shape of Dose Response Curve

If a signal molecule X controls a target molecule Y, either directly or indirectly, then the steady-state transfer function from X to Y can be quantitatively described as the ratio of the fractional change in Y over the fractional change in X, i.e.,

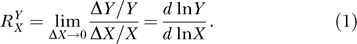




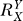
 , known as the response coefficient in MCA [16,19], and logarithmic gain in BST [17,20], is analogous to the gain of an amplifier or a transducer in electronics (in the rest of the text, we use the terms gain and response coefficient interchangeably). Assuming that 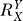 remains constant within a range of X of interest, then for some constant *k,*

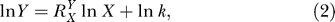
which is a linear function on a logarithmic scale, with 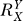 being the slope and ln*k* the intercept (Figure S1A). When transformed to a linear scale, Equation 2 becomes

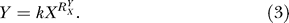



Hence, response Y is a single-term polynomial function of dose X of degree 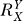 . The value of 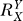 , relative to unity, determines the curvature of the Y versus X dose response curve (Figure S1B). Specifically, for 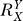 = 1, *Y* = *kX*, the Y versus X dose response is linear; for 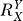 > 1, the dose response is sublinear (concave upward), denoting an ultrasensitive response; for 0 < 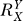 < 1, the dose response is superlinear (concave downward), denoting a subsensitive response. In situations where X negatively regulates Y, hence 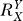 < 0, 1/Y versus X relationship observes the same curvature rule as above, which depends on |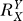 |. This logarithmic to linear transformation and the shape of dose response curve with respect to response coefficient has been previously described with the S-system in BST [17,21].

### Dose Response in Negative Feedback Regulation with Constant Local Gains

**Figure 2:**
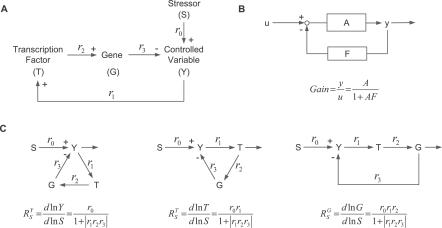
Analogy between the Anti-Stress Gene Regulatory Network and Proportional Negative Feedback Control System (A) The generalized anti-stress gene regulatory system. Stressor S increases production of controlled variable Y, which is cleared by gene product G; Y activates transcription factor T, which upregulates gene expression of G; *r*_0_ − *r*_3_ are local gains. (B) Proportional negative feedback system and its closed-loop gain. A is the open-loop gain, F is the feedback gain, and AF is the loop gain. (C) The feedback system in (A) is rearranged so that S is the input, and the species of interest (Y, T, and G) is positioned as the output. The systems-level gain for each of the species can be generalized in terms of the open-loop gain (*r*_0_, *r*_0_ *r*_1_, *r*_0_ *r*_1_ *r*_2_ for Y, T, G, respectively) and loop gain |*r*_1_ *r*_2_ *r*_3_|, conforming to the closed-loop gain in (B).

In this section, we set out to investigate the steady-state dose response relationships for the generalized negative feedback control scheme (Figure 2A; for model details see Figure S2 and Tables S1–S3). We defined that stress signal S increases the production rate of controlled variable Y with a local gain *r*_0_. Y then activates transcription factor T with a gain *r*_1_. T induces gene expression of enzyme G with a gain *r*_2_. Finally, G catalyzes the clearance of Y with a gain *r*_3_. Since G negatively regulates Y, the local gain *r*_3_ has a negative value. The total stress S is composed of S_0_ and S_e_ (S = S_0_ + S_e_) where S_0_ is the background stress level at the basal condition, and S_e_ is the stress level introduced by external stressors. The total stress level S is expressed as multiples of S_0_. Levels of Y, T, and G are also normalized to their respective levels at the basal condition where S = S_0_. For simplicity, we first considered the circumstance where all local gains are independent of each other and remain constant as the value of S is varied.

According to signal transfer/modular response analysis [22–24], the systems-level gain for Y, T, and G over S can be mathematically derived (see Text S1 for derivation) and expressed as follows:

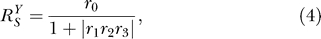


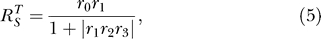


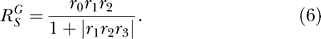



After rearranging the feedback control graph in Figure 2A by taking S as the input, and Y, T, and G as the respective output (Figure 2C), Equations 4–6 can be uniformly expressed in the format of

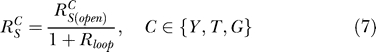
where 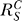 and 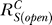 are, respectively, the systems-level (closed-loop) gain and open-loop gain for C over S, and *R_loop_* = |*r*_1_ *r*_2_ *r*_3_| is the loop gain. This formalism, conforming to that originally derived for intracellular signal propagations with feedback [17,20,22,24], is analogous to the closed-loop gain of a proportional feedback control system such as an electronic amplifier (Figure 2B).

The shapes of dose response curves, i.e., linear, superlinear, or sublinear, for the control circuit in Figure 2A depend on the systems-level gains. Assuming the production rate of Y is proportional to stress level S, and Y is cleared at a first-order rate by G (i.e., level of Y is far from saturating G), then *r*_0_ = 1, i.e., the controlled variable Y increases linearly with S in the absence of the feedback. Equations 4–6 can be simplified to:

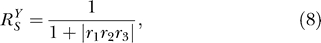


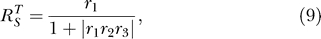


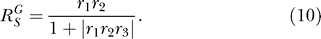



According to Equation 8, for the controlled variable Y, 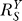 is always less than or at best equal to unity since the loop gain *R_loop_* ≥ 0 (zero is equivalent to open loop). Therefore, the Y versus S dose response curve is superlinear or at best linear. The smaller 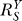 is, the more superlinear the dose response curve becomes, and Y is more insensitive to changes in S. Since the goal of the feedback gene regulation is to maintain homeostasis for Y (which could be ROS, DNA adduct, misfolded protein, etc.), it is desirable to have *R_loop_* as large, hence 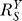 as small as possible, to effectively resist perturbations.

**Figure 3:**
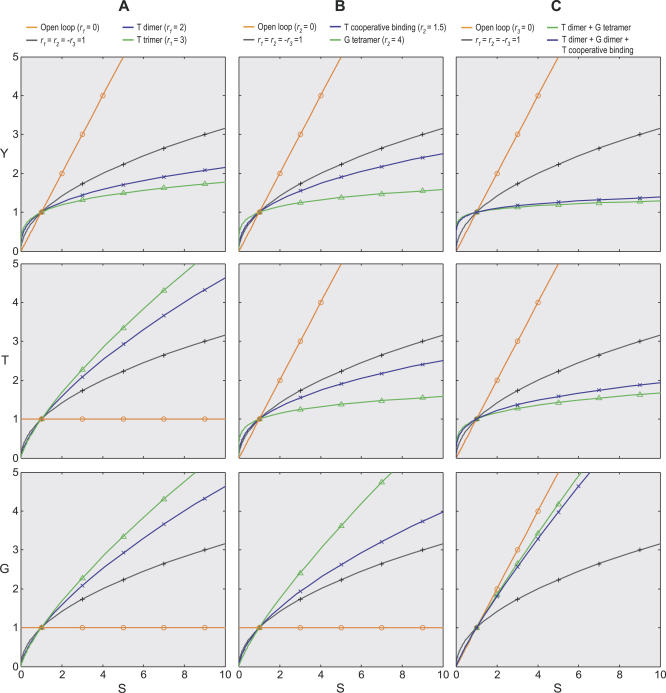
Effects of Local Gains on Shape of Dose Response Curves in the Anti-Stress Gene Regulatory Network in Figure 2 (A) Enhancing local gain *r*_1_ through dimerization or trimerization of transcription factor T increases the superlinearity of Y versus S dose response curve, decreases the superlinearity of T versus S and G versus S dose response curves. (B) Enhancing local gain *r*_2_ through T cooperative binding to the gene promoter or tetramerization of gene product G increases the superlinearity of Y versus S and T versus S dose response curves, decreases the superlinearity of G versus S dose response curve. (C) Effects of combinatorial changes in *r*_1_ and *r*_2_; green line (*r*_1_ = 2, *r*_2_ = 4), blue line (*r*_1_ = 2, *r*_2_ = 2×1.5); note that high loop gains tend to linearize the G versus S dose response curve. Default local gains: *r*_1_ = *r*_2_ = −*r*_3_ = 1. Solid line, simulation results; symbol, analytical results using Equations 8-10.

Augmentation of loop gain *R_loop_* can be achieved by increasing local gain *r*_1_, *r*_2_, and *r*_3_, either alone or in combination. Along the feedback loop—from activation of transcription factor, to gene induction, to enzyme formation, to enzymatic reaction—a variety of local interactions can operate in an ultrasensitive manner to provide high local gains (ultrasensitivity is generally defined as a response that has a Hill coefficient greater than unity). For instance, in response to stress, some transcription factors involved form homodimers or homotrimers to become transcriptionally active [25,26]. Ideally, homodimerization and homotrimerization can give *r*_1_ a characteristic value of 2 and 3, respectively. Simulation results indicated that dimerization or trimerization of T increasingly suppresses the Y versus S dose response curve, consistently matching the analytic results (Figure 3A, top panel). For local gain *r*_2_, it can be enhanced at least by the following two mechanisms. For one, a transcription factor may interact with promoters of inducible genes cooperatively if multiple copies of its response element exist. This cooperativity in DNA binding results in a more repressed Y versus S dose response curve in our control scheme (Figure 3B, top panel, blue line). For another, many enzymes induced in response to cellular stresses need to form homodimers or even tetramers from their initial translated products to become fully active. For instance, enzyme glutathione peroxidase (GPx), induced by oxidative stress and responsible for removing H_2_O_2_ and lipid peroxide, is a tetramer [27]. Similar to dimerization of transcription factors, these multimerization processes enhance local gain *r*_2_, leading to a more robust homeostatic control of Y (Figure 3B, top panel, green line). As far as *r*_3_ is concerned, which is the local response coefficient of Y controlled by G, no obvious interactions appear to be able to specifically enhance it. And since an enzyme generally has a linear control over its reaction rate (i.e., the elasticity is unity [15]), a characteristic value of −1 would be expected for *r*_3_ under conditions where G is far from being saturated by Y (how S and Y level influences *r*_3_ will be considered in later sections). Lastly, cells are likely to use combinations of ultrasensitive steps described above to achieve a large loop gain for robust homeostasis. For instance, dimerization of T, coupled with cooperative binding and dimerization of G, gives rise to a very insensitive, almost horizontal Y versus S dose response curve (Figure 3C, top panel, blue line). In contrast, when the feedback loop is broken, by opening up at either of the local steps, the Y versus S dose response curve is linearized (Figure 3, top panels, orange lines).

The shape of dose response curves for gene expression of G is determined by the systems-level gain 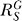 according to Equation 10. In contrast to the Y versus S dose response, which is superlinearized by increases in local gain *r*_1_ and *r*_2_, similar increases in *r*_1_ and *r*_2_ tend to linearize the G versus S dose response curve (Figure 3, bottom panels). This is because when the loop gain *R_loop_* ≫1, Equation 10 can be simplified to 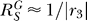 . As previously discussed, 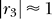 for relatively small Y, hence 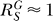 , indicating a linear relationship. This sole dependency of 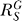 on feedback gain *r*_3_, and linearization of the output, as suggested by Savageau with BST [17,20], is similar to that of an operational amplifier with a very high open-loop gain. On a similar basis, it has been shown that mitogen-activated protein kinase (MAPK) signaling cascade can potentially ultrasensitize or linearize its output by adjusting the negative feedback strength [28,29]. The shape of dose response curves for transcription factor T is determined by the systems-level gain 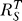 according to Equation 9. Local gain *r*_1_ and *r*_2_ alter the curvature of the dose response curve in opposite directions—increases in *r*_1_ reduce the superlinearity, whereas increases in *r*2 further superlinearize the T versus S dose response curve (Figure 3, middle panels).

### Dose Response in Negative Feedback Regulation with Varying Local Gains

In the above section, we explored in principle the shape of dose response curves in a homeostatic gene regulatory system with negative feedback, and how the curvature is altered by local gains distributed in the feedback loop. In these analyses, local gains were independent of each other and remained at their respective characteristic values as S varied. In an actual gene regulatory network, local gains at different steps and the total loop gain are unlikely to remain constant in response to a wide range of S. The resulting dose response curves are typically more complex than a simple linear, superlinear, or sublinear function can describe. Despite such complexity, it is possible to decompose a dose response curve into distinct phases each associated with a specific profile of changes in local/loop gains. In this section we set out first to consider the shape of each individual phase in isolation, and then to reconstruct the full-range dose response curve by linking individual phases in the order they are expected to become active as S increases.

#### Recovery of local gains from constitutive activation-imposed gain repression.

**Figure 4:**
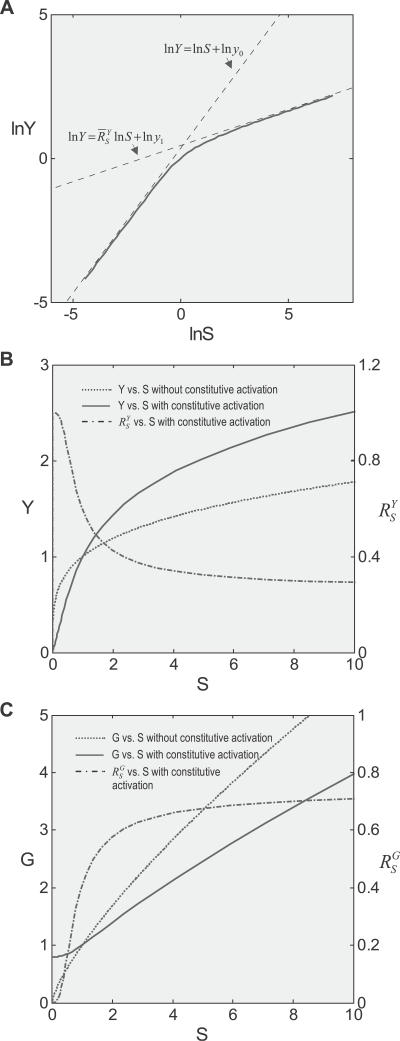
Effect of Constitutive Activation on Systems-Level Gains and Dose Response Curves Constitutive activation was modeled by implementing a basal-level expression of G in addition to T-driven expression. (A) The ln*Y* versus ln*S* curve transitions from a linear function ln*Y* = ln*S* + ln*y*_0_ to ln*Y* =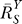 ln*S* + ln*y*_1_ in the presence of constitutive activation. (B) In the presence of constitutive activation, systems-level gain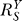 (dash-dotted line) decreases from unity to asymptotically approach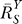 ; in the absence of constitutive activation,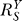 remains at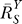 (unpublished data). Y responds to S in a more sensitive or less controlled manner in the presence of constitutive activation (solid line) than in its absence (dotted line). (C) In the presence of constitutive activation, systems-level gain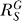 (dash-dotted line) increases from zero to asymptotically approach a maximum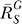 ; in the absence of constitutive activation,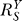 remains at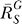 (unpublished data). G responds to S in a more sluggish manner in the presence of constitutive activation (solid line) than in its absence (dotted line).

In a feedback control scheme such as in Figure 2A, in addition to being activated by its upstream species with a characteristic response coefficient, each downstream species in the feedback loop may have an independent basal constitutive activity. For instance, gene G may be constitutively expressed at a certain level even in the absence of transcription factor T. With T controlling G on top of this basal level, the actual value of local gain *r*_2_ varies as T drives the expression of G to higher levels. With small S and therefore small T, constitutive expression of G dominates, rendering the overall expression of G insensitive to changes in T, hence a small *r*_2_. As S and T increases, T-induced expression of G will gradually surpass the constitutive expression and become dominant. In this process, *r*_2_ steadily recovers to approach a maximal level (i.e., the characteristic local response coefficient of G controlled by T in the absence of constitutive expression). Local gains in other steps in the feedback loop may undergo a similar recovery from repression owing to constitutive activities. Such slow increases in local gains in approaching their respective characteristic values lead to a similar sluggish increase in the loop gain *R_loop_*. As a result, the systems-level gain 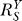 for Y (Figure 4A and dash-dotted line in Figure 4B) begins with unity, then decreases to asymptotically approach a fixed value 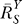 . The corresponding Y versus S dose response (Figure 4B, solid line) is characterized by a curve transitioning from an initial linear function of *Y* = *y*_0_ *S* for very small S to a superlinear function of 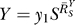 for very large S. And compared with the situation devoid of constitutive activation (Figure 4B, dotted line), the dose response in its presence, though superlinear in appearance, does not bend downward as much, indicating a less controlled stage of stress response. For G, the systems-level gain 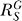 (Figure 4C, dash-dotted line) increases from zero, asymptotically approaching a fixed value 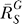 . The G versus S dose response (Figure 4C, solid line) is characterized by a curve transitioning from a horizontal line *G* = *g*_0_, through a transient sublinear stage, to function 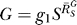 . And compared with the situation devoid of constitutive activation (Figure 4C, dotted line), gene expression in its presence is sluggish.

#### Decreasing *r*_1_ and *r*_2_ due to saturable activation.

**Figure 5:**
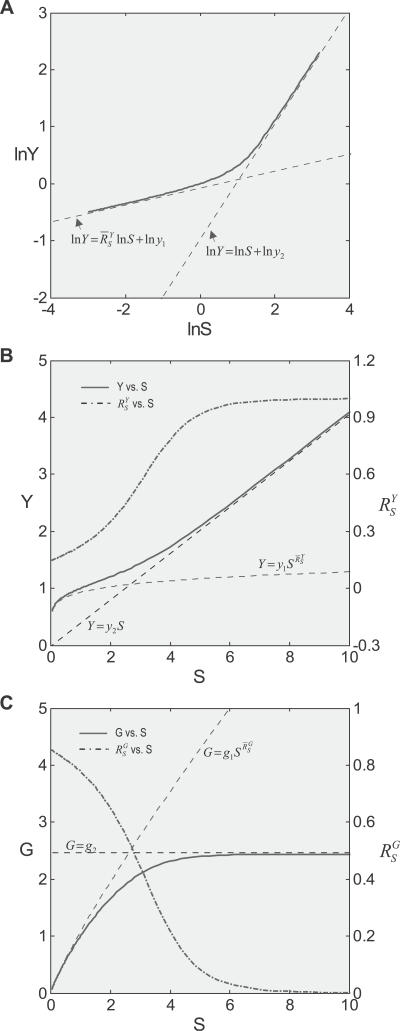
Effect of Saturation of Gene Activation on Systems-Level Gains and Dose Response Curves Saturation of gene activation was modeled by implementing saturable T binding to the gene promoter. (A) In the presence of saturation of gene activation, the ln*Y* versus ln*S* curve transitions from a linear function ln*Y* =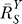 ln*S* + ln*y*_1_ to ln*Y* = ln*S* + ln*y*_2_. (B) Systems-level gain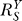 (dash-dotted line) increases from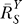 to asymptotically approach unity. The Y versus S curve (solid line) transitions from a superlinear function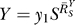 , through a sublinear segment, to a linear function *Y* = *y*_2_ *S*. (C) Systems-level gain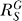 (dash-dotted line) decreases from the maximum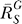 to asymptotically approach zero. The G versus S curve (solid line) transitions from a function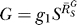 to a horizontal line *G* = *g*_2_.

After recovering from gain repression by constitutive activation or in its absence, each local step operates at its characteristic gain value. However, it is unlikely that local gains would remain at these values for very high input of S. The saturable nature of biochemical interactions sets an upper limit for the degree of activation. For instance, if the transcription factor is activated by phosphorylation, then the abundance of the phosphorylated form cannot be greater than the total amount of that transcription factor. With respect to promoter binding, once the concentration of the specific transcription factor becomes much greater than the dissociation constant (K_d_), the percentage binding will increase little even if the transcription factor continues to rise in concentration. In the process of approaching saturation of activation, local gains fall from their characteristic values toward zero. Accordingly, the loop gain *R_loop_* decreases from an initial value to zero, as well. For the controlled variable Y, the systems-level gain 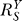 would increase, according to Equation 8, from an initial value 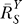 to unity (Figure 5A and dash-dotted line in Figure 5B). The resulting Y versus S dose response (Figure 5B, solid line) is characterized by a curve transitioning from an initial superlinear function 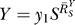 for small S to a linear function *Y* = *y*_2_ *S* for large S, interposed with a transitional sublinear segment. In the process of approaching saturation, the systems-level gain 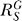 for G (Figure 5C, dash-dotted line) decreases from a fixed value 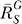 to zero. Accordingly, the G versus S dose response starts with a function of 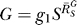 , then plateaus as 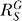 approaches zero (Figure 5C, solid line). In a sense, once gene activation is saturated, the control scheme degenerates to an open-loop system.

#### Increasing *r*_0_ and *r*_3_ due to saturation of G by Y.

The steady-state concentration of the controlled variable Y is determined by its production and clearance rates. In the generalized control scheme, Y is produced at a rate of *k*_0_ *S,* and removed by enzyme G at a Michaelis–Menten rate of *k_c_GY*/(*K_m_* + *Y*). Then, the local gain

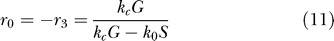



**Figure 6:**
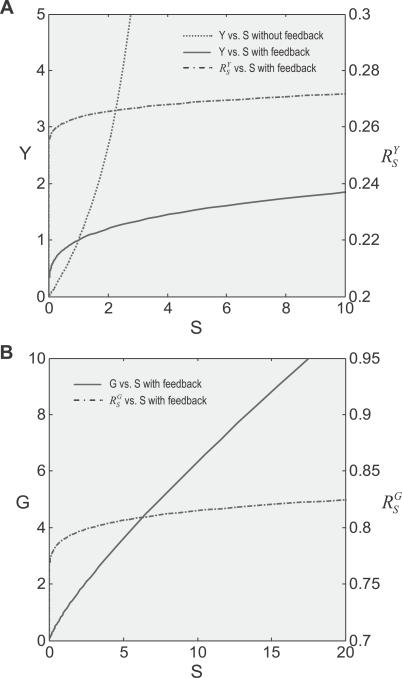
Effect of Saturation of Enzyme G Activity by Controlled Variable Y on Systems-Level Gains and Dose Response Curves (A) In the absence of feedback control, saturation of G by Y causes a sublinear catastrophic increase in Y in response to S (dotted line); in the presence of feedback control, the Y versus S dose response curve (solid line) grows in a much suppressed, superlinear fashion; the corresponding systems-level gain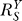 is low and varies only slightly as suggested by Equation 12. (B) The enzyme G versus S dose response curve (solid line) grows in a nearly linear fashion, as the systems-level gain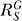 increases to approach unity according to Equation 14.

Consistent with the early assumption, *r*_0_ = −*r*_3_ ≈ 1 for small S. As S increases, *r*_0_ rises from unity to infinity because the enzymatic activity of G is eventually saturated by Y. In the absence of negative feedback, the controlled variable Y will first increase linearly with S, then sublinearly with an accelerated gain (Figure 6A, dotted line); as *k*_0_ *S* surpasses *k_c_G,* the system becomes unstable. Such an accelerated rise in Y at the late stage could be catastrophic to cells because a small increase in S causes an amplified elevation in Y. In contrast, with the negative feedback loop in place, the systems-level gains described by Equation6 4-6 can be reformatted to

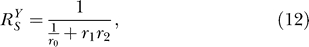


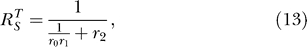


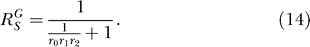



Thus, as long as *r*_1_ *r*_2_ remains relatively large, i.e., gene activation is still at work, engagement of the negative feedback permanently suppresses the otherwise catastrophic response of Y, with the systems-level gain 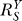 at 1/(1+ *r*_1_ *r*_2_) for small S and 1/*r*_1_ *r*_2_ for large S. Correspondingly, the Y versus S dose response will migrate from a superlinear to a less superlinear curve (Figure 6A, solid line), and the G versus S dose response from a superlinear to a nearly linear curve (Figure 6B, solid line). Notably if *r*_1_ *r*_2_ is sufficiently large, these curvature changes could be negligible. It should also be noted that although Equation 11 was derived using a simple Michaelis–Menten kinetics, the equivalence between *r*_0_ and −*r*_3_ and the generally increasing trend of these gains as S increases hold true for other enzymatic kinetics, such as reversible reactions and multisubstrate reactions (derivation not shown), given the fact that in most cases an enzyme's elasticity is unity [15].

#### Composite dose response curves.

**Figure 7:**
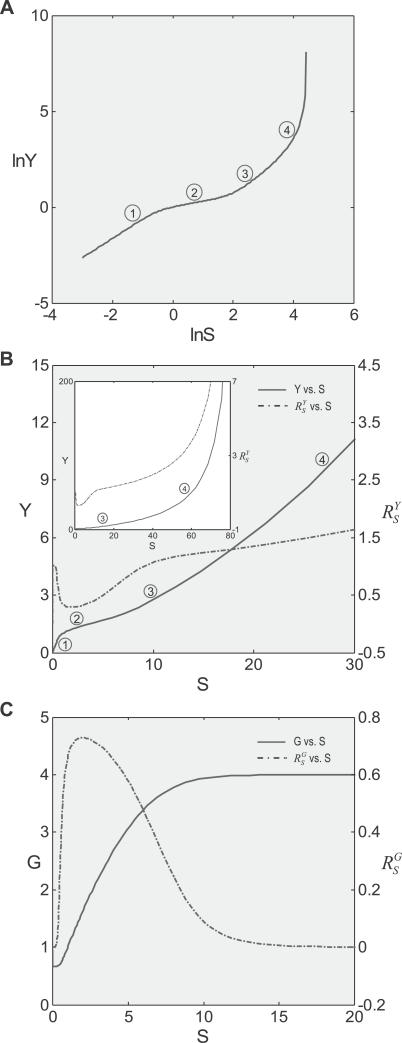
Multi-Phasic Dose Response Relationships and Systems-Level Gains in the Presence of Constitutive Activation, Saturation of Gene Activation, and Saturation of Enzyme G by Y Phase 1: superlinear with lesser control; phase 2: superlinear more highly controlled; phase 3: linear uncontrolled; phase 4: sublinear catastrophic.

Above, we have discussed how individual gain-changing events associated with constitutive activation, saturation of gene activation, and saturation of G-catalyzed reaction, generate different types of curvature changes in dose response relationships. In a homeostatic gene regulatory network, it is unlikely that these events take place exclusively of one another. Rather, these processes could come into play in combinations and in orders as the stressor level increases, steering the dose response curve through distinct phases. A very possible scenario which can fully manifest all the effects of these events is as follows (Figure 7). In the beginning when S is very small, the gain-repressing effect of constitutive activation keeps the loop gain *R_loop_* very small, hence 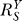 is close to unity. As S increases, the influence of constitutive activation lessens, bringing 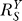 closer to the lowest possible value. In this initial 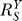 -decreasing phase, the Y versus S dose response becomes increasingly superlinear, but compared with situations where no constitutive activation exists, Y is less controlled. The second phase, which is also superlinear in appearance, is characterized by 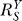 remaining at the lowest value, and is a stage where Y is least sensitive to changes in S compared with other phases. As S increases further, signaling events leading to gene activation move closer to saturation; 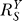 thus starts to rise and approaches unity as saturation of activation fully sets in. In this process, the superlinear second phase first bends upward through a transitional sublinear segment, then extends into the third phase, which is largely linear. As discussed in the next section, the sublinear transition from phase 2 to 3 could play a significant part in the shape of the curve in the low-dose region. At the end of the third phase, saturation of G by Y becomes increasingly significant, the dose response curve extends into the catastrophic fourth phase, which is sublinear in appearance and grows with increasing 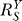 . The corresponding G versus S dose response initially experiences a transient unresponsive phase, followed by a superlinear or nearly linear phase, which eventually levels off into a plateau phase (Figure 7C).

**Figure 8:**
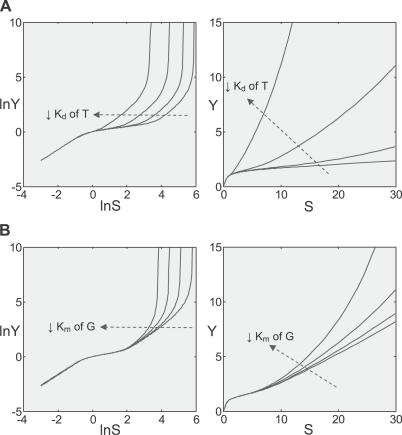
Variation of Saturation Terms Affects the Length of Superlinear Controlled and Linear Uncontrolled Phases (A) Lowering the dissociation constant K_d_ for transcription factor T binding to the gene promoter shortens the superlinear highly controlled phase. (B) Lowering the Michaelis–Menten constant K_m_ of enzyme G for clearance of Y shortens the linear uncontrolled phase.

Although in theory the Y versus S dose response curve could consist of at least four phases, the actual occurrence and length of each phase depends on whether the required individual gain-changing events exist and how far they are separated from each other in terms of level of S. If little or no basal constitutive activation exists, the initial superlinear phase will be minimal. The span of the superlinear second phase depends on how soon the signaling events leading to gene activation saturate. Advancing the gene saturation event by, for instance, lowering the K_d_ value for transcription factor T binding to the gene promoter, shortens the second phase (Figure 8A). The span of the linear third phase depends on the distance of separation between saturation of gene activation and that of clearance of Y by enzyme G. Simulation results indicated that with smaller K_m_ for G, which brings the latter gain-changing event closer to the former, the linear phase increasingly shortens, and eventually plays little role in the overall shape of the curve (Figure 8B).

#### Curvature in the low-dose region.

**Figure 9:**
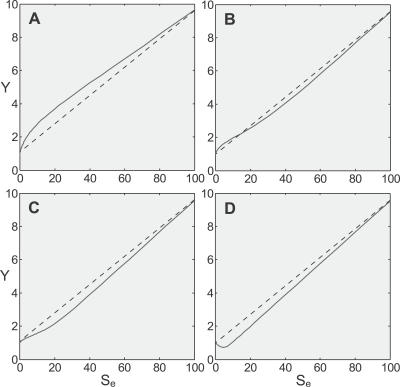
Variations in the Pre-Transcriptional Local Gain (*r*_1_), Level of Constitutive Activation, Earliness of Saturation of Gene Activation, and Degree of Feedforward Activation Can Qualitatively Alter the Curvature of the Y versus S_e_ (External Stress) Dose Response Curve in the Low-Dose Region (A) Relatively small *r*_1_, high constitutive activation, and large K_d_ tend to retain the superlinear appearance in the low-dose region. (B) Intermediate conditions lead to minimal curvature change. (C) Relatively large *r*_1_, small constitutive activation, and small K_d_ render a sublinear appearance in the low-dose region. (D) Introduction of feedforward activation can depress Y initially, giving rise to a J-shaped dose response. Note: solid lines are simulation results, dashed straight lines were drawn to show how linear extrapolation from the high-dose region to the basal level can mis-estimate the response at low doses. Saturation of gene activation was modeled by changing K_d_ for transcription factor T binding to the gene promoter.

A common practice in assessing the biological risk for exposure to low-dose *external* stressors is to extrapolate linearly from high doses, where the impact can be reliably measured, to the background risk level at basal conditions (where S = S_0_). The underlying assumption is that the dose response behaves consistently in a linear fashion from high- through low-dose regions. Yet in the absence of the knowledge of low-dose curvature and its relationship with high-dose responses, linear extrapolation is unlikely to accurately represent low-dose risks. As we demonstrated above, in a homeostatic cellular defense system the low-dose region is intrinsically nonlinear. Notably, the dose response curve for Y versus external stress S_e_ in the low-dose region is composed primarily of the superlinear phases and the transitional sublinear segment linking phase 2 and 3 (note: in constructing the dose response curve for Y versus the *external* stress S_e_, the background stress level S_0_ needs to be subtracted from the total stress S, and this is simply done by shifting the dose response curve derived for S to the left by S_0_ amount, which is unity in this study). The primary curvature in the low-dose region depends on the relative influences from the superlinear phase and sublinear segment. Simulation results demonstrated that under conditions where the pre-transcriptional local gain (for instance *r*_1_) is relatively small, the effect of constitutive activation is not negligible, and gene activation does not saturate early, a superlinear appearance occupies the low-dose region (Figure 9A). In contrast, the reverse conditions render a sublinear curve to dominate the low-dose region (Figure 9C). Intermediate conditions lead to less dramatic curvature changes in either direction (Figure 9B). Furthermore, introduction of feedforward activation of anti-stress genes can depress concentration of Y initially, leading to a J-shaped nonmonotonic dose response in the low-dose region (Figure 9D, see Text S2 for details on feedforward activation).

### Anti-Electrophilic Stress Gene Regulatory Network

**Figure 10:**
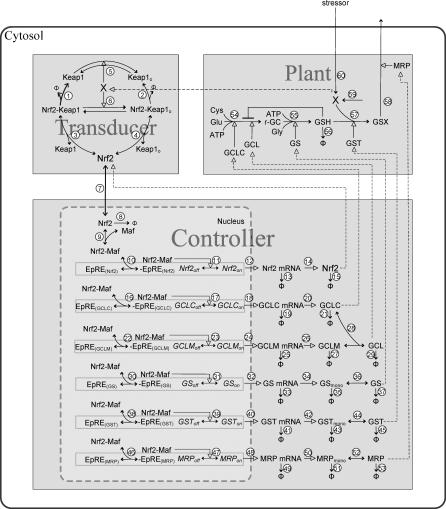
Schematic Diagram of the Anti-Electrophilic Gene Regulatory Network Model Refer to the main text for general description of the interactions, Text S4 and Tables S4 and S5 for kinetic details in the numbered reactions. Dashed lines with empty arrow head indicate the direction of logical control flowing between the transducer, controller, and plant. The diagram was generated in PathwayLab (InNetics, http://www.innetics.com).

The anti-stress gene regulatory network illustrated in Figure 2A is a generalized control scheme for cellular homeostasis. With respect to risk assessment, it is practically important to determine whether the dose response transition derived from this general scheme would hold in more complex and realistic anti-stress biological systems, which often involve multiple genes, enzymes, and biochemical reactions. Extensive validation of the proposed transition would require experimental studies systematically characterizing the full-range dose responses, especially at low doses with detailed and reliable measurements. Unfortunately, such studies are rare so far, and, in most cases, the inability to obtain low-dose data reliably and efficiently has been the primary motivation behind dose response extrapolation. Nevertheless, in keeping with the current idea, available experimental data in rat livers have shown that the level of DNA adducts in response to carcinogen dimethylnitrosamine (DMN) [30], and that of protein conjugates in response to electrophile-generating agent vinylidene chloride (VDC) [31], indeed followed the proposed dose response transition (Text S3). To further solidify our conclusions, in the remaining section we focused specifically on the mammalian anti-electrophilic stress system. By formulating a detailed model of this system (Figure 10), we studied its dynamic and dose response behaviors and how the complexity of the underlying gene regulatory network contributes to the system's controllability (for model details, see Text S4 and Tables S4 and S5).

Electrophiles are electron-attracting chemical agents/metabolites that are cyto- or genotoxic via reactivity with proteins and DNAs. They are primarily detoxified in the cell by conjugation with reduced glutathione (GSH) enzymatically or in some cases non-enzymatically. The anti-electrophilic stress gene regulatory network in mammalian cells can be decomposed, from an engineer's perspective, into three functional units as seen in a classical control system, i.e., transducer, controller, and biochemical plant (Figure 10). The transducer contains Kelch-like ECH-associating protein 1 (Keap1) and nuclear factor erythroid 2-related factor 2 (Nrf2), which sense the levels of intracellular electrophile (X) and ROS. Specifically, Keap1 is a cytosolic cysteine-rich protein that facilitates the degradation of transcription factor Nrf2 through ubiquitination [32,33]. An increase in the level of intracellular electrophiles causes conjugation and/or oxidization of certain key cysteine residues in Keap1, rendering Keap1 incapable of mediating Nrf2 ubiquitination and degradation [34,35]. The ensuing stabilization of Nrf2 results in elevated cytosolic Nrf2 levels through de novo protein synthesis and subsequently its nuclear translocation. The controller receives the input from Nrf2 through the electrophile response element (EpRE) and integrates it with other transcriptional signals to regulate gene expression of a set of anti-electrophilic enzymes, including glutamate cysteine ligase catalytic subunit (GCLC), glutamate cysteine ligase modifier subunit (GCLM), glutathione synthetase (GS), glutathione S-transferase (GST), and multidrug resistance-associated protein (MRP) [36–40]. These enzymes function in cohort to control the level of electrophiles by catalyzing a set of interconnected metabolic reactions in the biochemical plant. Specifically, GCLC, holoenzyme GCL, (heterodimer of GCLC and GCLM), and GS collectively contribute to the de novo synthesis of GSH in two sequential reactions. GST then transforms, using GSH as a co-substrate, the electrophile into less toxic and more water-soluble glutathione conjugates (GSX). GSX is then extruded by MRP out of the cell.

**Figure 11:**
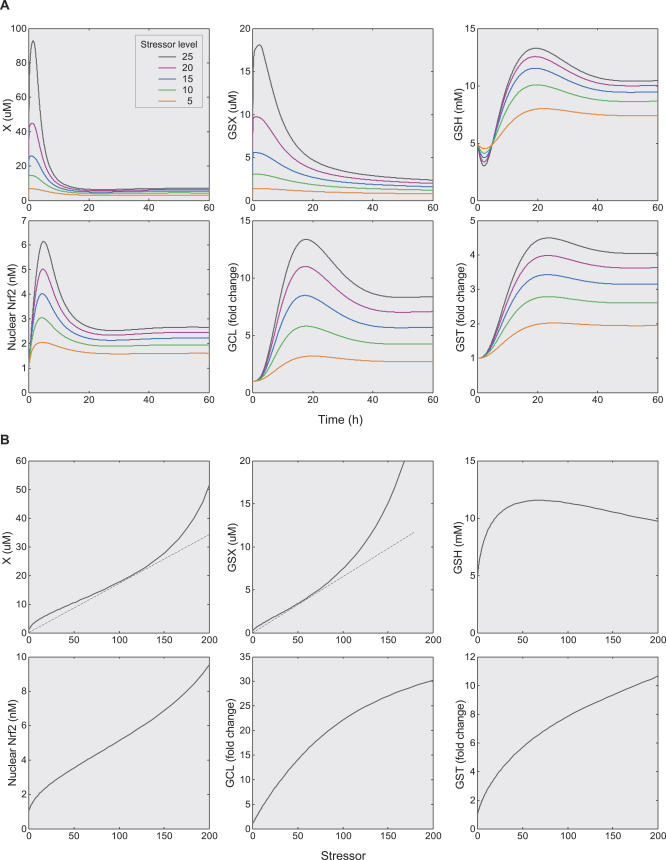
Simulation Results for the Anti-Electrophilic Gene Regulatory Network Model (A) Temporal changes in the levels of electrophile X, conjugation product GSX, GSH, Nrf2, GCL, and GST, in response to different stressor doses. (B) Steady-state dose response curves for the molecular species listed in (A). Dashed tangent lines originating from (−1, 0) were drawn to help visualizing curvature changes. One unit of external stressor level is equivalent to producing X at the basal production rate.

Our simulation indicated that the anti-electrophilic defense system launches a typical adaptive response when challenged continuously with electrophilic stresses (Figure 11A). The simulated dynamics is similar to experimental observations in a variety of cells exposed to many electrophiles, such as 4-hydroxy-2-nonenal (4-HNE) and 15-deoxy-delta(12,14)-prostaglandin J2 (15d-PGJ_2_) [41,42]. The electrophile X initially rises sharply, but after a few hours it settles at much lower steady-state levels. GSX follows a similar dynamic change, albeit it declines to steady states more slowly. In comparison, Nrf2, GCL, GS (unpublished data), and GST levels peak in a more delayed manner before leveling off. MRP, due to its long half-life (27 h in the current model), does not reach a steady state until a much later time (unpublished data). The level of intracellular GSH initially decreases as a result of consumption by electrophiles. But the downtrend is soon reversed as the expression level of GCL and GS increases. After a few hours, GSH surpasses its basal level and then peaks before settling down on elevated steady-state levels.

The steady-state dose response curve for electrophile X, which is a controlled variable here, transitions from an initial superlinear controlled phase, through a minimal linear phase, to a sublinear catastrophic phase (Figure 11B). This transition is clearly consistent with the dose response profile derived from the generalized control scheme. Conjugation product GSX, a minor controlled variable here due to its much lower toxicity, also experiences an initial superlinear phase, albeit of much less extent, before moving upward sublinearly. Compared with X, the smaller superlinear controlled phase of GSX is likely due, at least in part, to the fact that MRP is the only gene controlling GSX. The steady-state GSH dose response has a biphasic appearance—it first increases at small doses then decreases as the dose increases further. This biphasic profile has been observed in human epithelial cells treated with lipid electrophile 15-deoxy-delta(12,14)-prostaglandin J2 (15d-PGJ_2_) [43]. Interestingly, nuclear Nrf2 appears to have a dose response profile similar to X, suggesting the transducer containing Keap1 may have a close-to-linear signal transfer property. Levels of enzymes such as GCL and GST increase dose dependently and tend to plateau at high doses.

As far as homeostatic feedback control is concerned, the mammalian anti-electrophilic system, as at least in the current model, is complex in the following aspects. First, control of detoxification of electrophile X is a concerted action by multiple genes through a series of metabolic reactions, including co-substrate GSH synthesis by GCL and GS, conjugation of X by GST, and extrusion of GSX by MRP. Second, there are several places in the feedback loop where local gains can be enhanced for effective homeostatic control. These include formation of GS, GST, and MRP homodimers by their respective monomeric subunits [44–46], formation of holoenzyme GCL heterodimers by GCLC and GCLM [36], and positive auto-regulation of Nrf2 [47,48]. Third, the core electrophile-detoxifying reaction catalyzed by GST is, in many cases, subjected to product inhibition by GSX [49–51]. This inhibition suggests that extrusion of GSX and regulation of MRP, though downstream of X, may also play a role in its homeostatic control. In the following section, we investigated how individual elements of these complexities affect the homeostatic performance of the control system.

**Figure 12:**
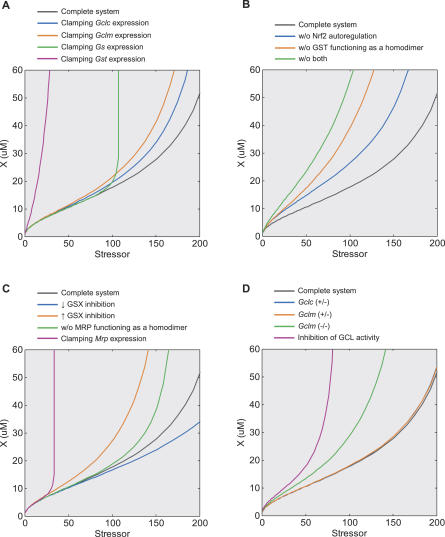
Dose Response Curves of Electrophile X versus Stressor under Various Disrupted Conditions (A) Effects of deregulation of *Gclc, Gclm, Gs,* and *Gst* genes by Nrf2. Deregulation was implemented by clamping mRNAs of respective genes at levels seen at the basal condition. (B) Effects of lack of Nrf2 autoregulation and/or GST functioning as a homodimer. Removal of Nrf2 autoregulation was implemented similarly as in (A); de-dimerization of GST was implemented by replacing the quadratic term in Reaction 44 with a linear term that left GST concentration at the basal condition unchanged. (C) Effects of product inhibition of GST-catalyzed reaction. For reduced GSX inhibition, Ki in Reaction 57 was increased to 850 μM from the default value 85 μM; for increased GSX inhibition, Ki was lowered to 8.5 μM. Deregulation and de-dimerization of MRP were similarly implemented as above for other enzymes. (D) Effects of alteration in GSH levels via gene disruption or GCL activity inhibition. Genetic disruption of *Gclc* and *Gclm* genes was implemented by either setting the respective genes to half of the default values for heterozygous deficiency or to zero for homozygous deficiency. GCL activity inhibition was implemented by lowering k_c_ in Reaction 54 to 2.5% of the default value.

According to the two-gene control system analyzed in Text S5, inclusion of each additional feedback gene regulation adds to the loop gain (Equation S8). To examine the role of each individual gene regulation in the controllability of electrophile X, simulations were performed by clamping their expression levels at values seen at the basal condition. The results revealed that deregulation of genes responsible for GSH synthesis, i.e., *Gclc, Gclm,* and *Gs,* does not affect the controllability at low stressor levels significantly, as X level follows very closely that in the complete system (Figure 12A). However, the response diverges at intermediate stressor levels, swinging upward sharply with clamped *Gs* expression, but more mildly with clamped *Gclc* or *Gclm* expression. Regardless, the general superlinear-to-sublinear dose response profile seems to hold in these cases. In contrast, deregulation of *Gst* results in a sharp early rise in X level such that it enters the catastrophic phase at very low doses (Figure 12A, red line). Clamping *Mrp* gene expression resulted in an initial superlinear response that overlaps with that for the complete system, but it soon shoots upward almost vertically as the dose increases further (Figure 12C, red line). In summary, it appears that to keep the electrophile level contained, it is more crucial to adaptively upregulate *Gst* and *Mrp* than to enhance GSH replenishment by upregulating *Gcl* and *Gs*. Moreover, the simulation supports the concept that transcriptional feedback regulation of multiple anti-stress enzymes enhances the loop gain and improves the resistance to cellular stressors, albeit each individual gene regulation may exert its effect at different dose levels.

We next investigated the homeostatic role played by processes potentially enhancing local gains. Simulations indicated that in the absence of certain gain-enhancing processes, such as GST homodimerization, Nrf2 autoregulation, or both, the dose response curve for X diverges upward at low doses with an earlier onset of the catastrophic sublinear phase (Figure 12B). In the case where MRP can function without forming a dimer, the dose response curve for X mostly overlaps with that for the complete system until it diverges upward at intermediate doses (Figure 12C, green line). While exclusion of these gain-enhancing processes notably renders the system more sensitive to external perturbations of different levels, the superlinear-to-sublinear dose response profile remains intact in most cases. Simulating the system without GS dimerization does not alter the dose response curve for X when compared with the complete system (unpublished data), suggesting a lesser role of this process in the homeostatic control.

The GSH conjugates of many electrophiles can exert an inhibitory action on the catalytic activity of GST [49–51]. With product inhibition, the homeostatic control of X will be affected by the level of GSX and MRP that controls GSX extrusion. This effect was clearly demonstrated by altering the inhibition constant K_i_ of GSX over GST. An increase in K_i_ delays the occurrence of the catastrophic phase, whereas a decrease in K_i_ sensitizes the response, advancing the occurrence of the catastrophic phase (Figure 12C). As mentioned above, deregulation of *Mrp* gene expression (red line) and lack of MRP functioning as a homodimer (green line) both unfavorably affect the homeostatic performance, with the former being more damaging. Thus, through product inhibition, MRP, the enzyme located last in the detoxification chain, plays a significant role in the homeostatic control of X. In supporting this role, it has been experimentally demonstrated that MRP2 can potentiate GST A1–1 mediated cellular resistance to electrophilic agents [52,53]. All in all, the superlinear-to-sublinear dose response profile remains intact regardless of the alterations made to the system with respect to product inhibition.

The intracellular GSH level has been experimentally manipulated via both genetic and pharmacological means to study the role of GSH in anti-oxidant/electrophilic response [54–56]. Here we examined, in silico, how altered GSH levels via disruption of *Gclc* and *Gclm* genes, as well as inhibition of GCL activity, affect the homeostatic control in anti-electrophilic defense. Because mouse homozygous *Gclc*(−/−) knockout is embryonically lethal, it is not discussed here. While basal GSH level is 5 mM in the complete model system, it decreases to 4, 3.6, and 1.2 mM in our models, equivalent to *Gclc*(+/−), *Gclm*(+/−), and *Gclm*(−/−) knockouts, respectively. These values are in line with or close to changes in GSH levels reported experimentally for respective knockout mice, which are 80%, 43%~83%, and 9%~16% of that in the wild-type [54,55]. Our simulation revealed that both the *Gclc*(+/−) and *Gclm*(+/−) models produce a dose response curve for X that is almost identical to that with the complete system (Figure 12D). This unaltered anti-electrophilic control capability predicted with our model resonates with the fact that the heterozygous animals have nearly normal phenotypes and viability [54,55]. In contrast, more severe disruption of basal GSH levels, as observed with *Gclm*(−/−) homozygous knockout, results in a slightly elevated superlinear controlled phase followed by an earlier onset of the catastrophic phase (Figure 12D, green line). A similar dose response curve with a more advanced catastrophic phase was obtained by inhibiting GCL activity, which produces a basal GSH level of 0.5 mM in this case (Figure 12D, red line). Overall, these results are consistent with experimental findings that *Gclm*(−/−) knockout mice and cells depleted of GSH with GCL-inhibiting agent BSO are more sensitive to electrophilic/oxidative damage [54,56].

While most electrophilic compounds are detoxified through conjugating with GSH enzymatically, certain chemicals, such as the electrophilic intermediates of vinylidene chloride (VDC), can efficiently react with GSH to form conjugates without GST [57]. Thus, it is necessary to ascertain whether the dose response transition observed for electrophile X, which is conjugated enzymatically, will recur in nonenzymatic situations. Simulations indicated that although the dynamic responses are similar to GST-catalyzed situations (Figure S3A), lack of GST participation results in a less robust homeostatic control of X—the same stressor level produces a higher X level (Figure S3B). Nevertheless, the superlinear-to-sublinear profile seems retained, albeit less prominently. Interestingly, compared with GST-catalyzed situations, the homeostatic control of the minor controlled variable GSX becomes more robust and exhibits a more pronounced superlinear-to-sublinear appearance.

In summary, we have demonstrated that the dose response transition derived from the generalized control scheme holds well in an anti-stress gene regulatory network as complex as the system defending electrophilic stresses, which involves multiple genes, enzymes, and metabolic reactions. The transition profile is even retained in highly impaired circumstances, such as gene knockout and deregulation. The feedback regulation of multiple genes not only enhances the system's controllability, but also makes the system less vulnerable to functional disruptions of individual genes.

## Discussion

Cells do not remain passive when confronted with environmental challenges. To maintain a relatively stable intracellular milieu, they are equipped with a suite of specialized defense programs that are launched in response to various external stressors [2–5]. These defense mechanisms often comprise gene regulatory networks organized into negative feedback circuits, which can be decomposed into basic functional units seen in a classical control system. Our ultimate goal is to assess quantitatively the impact of external stressors, such as environmental toxicants, on such a complex control system and on consequent higher-level functions such as cell survival and death. To this end, it would be helpful to first reduce the complex systems to a basic control scheme and to study its behavior. The present study demonstrated that local gains, distributed in the feedback loop of a homeostatic gene network, shape the steady-state dose response curves, leading to linear, superlinear, or sublinear relationships under different conditions. The dose response relationship for intracellular controlled variables is multiphasic as stressor level increases—initial superlinear with lesser control, superlinear more highly controlled, linear uncontrolled, and sublinear catastrophic. The appearance of each phase depends on specific gain-changing events that come into play as the stressor level increases. Our work also indicated that responses in the low-dose region could vary from superlinear to sublinear, and even to J-shaped curvatures, depending on the strength of homeostatic regulation. Overall, these changing responses are consistent with the so-called dose-dependent transition proposed for many chemical compounds [58,59].

Analogous to the I/O relationship in a manmade control system implemented via proportional negative feedback, the steady-state systems-level gain in a gene regulatory network functioning to resist perturbations abides by a similar transfer principle. The systems-level gain for the controlled variable Y, transcription factor T, and gene product G can be generically described by their respective open-loop gain and the loop gain (Equation 7), a formalism that resembles the closed-loop gain of an electronic amplifier and conforms to that originally derived for intracellular signal propagations with feedback [17,20,22,24]. Under small S where *r*_0_ ≈ 1, the systems-level gain, or sensitivity of Y to S, 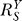 , is less than unity (Equation 8). This results in a superlinear Y versus S dose response curve. Since Y is the controlled variable, to minimize alterations in Y in response to changes in stress level S, it is desirable to keep 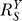 as small, and thus the loop gain *R_loop_* as large, as possible. The larger *R_loop_* is, the more superlinear the Y versus S dose response curve becomes, and cells are more resistant to perturbations. Since *R_loop_* is the product of individual local gains sequentially distributed along the feedback loop, increases in any individual local gain will augment *R_loop_*, leading to more robust homeostasis.

Delving into the molecular details of many anti-stress gene regulatory networks readily reveals that local gain enhancement appears to be a common strategy cells utilize for robust homeostasis. In this regard, cells are furnished with many biochemical reactions/interactions or functional modules that can transfer signals in an ultrasensitive, or even switch-like manner, and thereby enhance local gains. It is not uncommon that many transcription factors specifically involved in stress responses must homodimerize or homotrimerize to become transcriptionally active. For instance, in response to heat shock, heat shock transcription factor 1 (HSF1) monomers multimerize into homotrimers to gain affinity to bind the heat shock element (HSE) [25]. Ideally homodimerization and homotrimerization can enhance signal transfer sensitivity, thus the local gain, by a factor of 2 and 3, respectively. Binding of transcription factors to specific DNA response elements is the next step where the stress-triggered signal can be amplified. Existence of multiple copies of a response element in a gene promoter provides the possibility for cooperative binding, which is a classical interaction that can give rise to ultrasensitivity. In yeast, where the *Hsp82* promoter contains three copies of HSE in adjacency, HSF binds the promoter in a profound cooperative fashion to induce *Hsp82* gene expression [60]. Similar homeostatic roles played by transcription factor multimerization and cooperative binding have been suggested in maintaining intracellular protein concentrations [61,62]. Subsequent steps in the feedback loop, including RNA transcription and protein translation, are largely linear processes, adding little, if any, to local gains. But in many cases, the initial translation products need to form high-order multimers to become fully active enzymes. This process provides a gain-enhancing mechanism similar to transcription factor dimerization or trimerization. For instance, among the antioxidant enzymes that are activated in oxidative stress response, glutathione reductase and superoxide dismutase are homodimers [63,64]. More intriguingly, glutathione peroxidase (GPx) and catalase (CAT), the two major enzymes responsible for removing intracellular H_2_O_2_ and lipid peroxide, exist largely as homotetramers [27,65]. It is thus highly likely that apart from potentially stabilizing the enzymes, a primary function of dimer or tetramer formation is to augment the loop gain for robust redox homeostasis. To further improve signal transfer sensitivity, cells can also use localized positive feedback which is known to enhance response coefficient. A common positive feedback motif in the cell is gene autoregulation, in which a transcription factor upregulates gene expression of itself or its cofactors. In Nrf2-mediated gene regulatory network against electrophilic stress, the electrophile response element (EpRE) is found in *Nrf2* gene promoters, and Nrf2 can transcriptionally upregulate its own gene expression [47,48]. Lastly, a very common ultrasensitive signaling motif is the MAPK cascade, which can produce a switch-like response due to the combination of zero-order ultrasensitivity, distributive dual-phosphorylation, and layered arrangement [66]. Specifically, c-Jun N-terminal kinase (JNK), a member of the MAPK family, mediates a series of stress responses [67] and was shown to transfer signals in an ultrasensitive fashion [68]. Cells are likely to use combinations of these ultrasensitive mechanisms to enhance the loop gain as well as to compensate for gain losses from individual mechanisms operating at less ideal conditions, such as significant degradation of high-order multimers and substrate sequestration in zero-order covalent modification cycles [69].

Local gains do not remain characteristically constant as the feedback network is increasingly activated by external stressors. This stress level–dependent variation suggests that a dose response curve could undergo multiple phases that cannot be represented by a simple function. The slow recovery of local gains from repression [70], owing to constitutive activity of transcription factors or anti-stress genes, results in a sluggish response in gene expression, leaving the perturbation less countered under low-level stresses. Although such inadequacy in effectively mounting a protective response is seemingly undesirable, in certain situations it may be an energy-saving design. For cells living in an environment featuring frequent but minor fluctuations, they may have purposely evolved to tolerate perturbations of small magnitude to avoid otherwise expensive and frequent activation of anti-stress genes. Another situation where the less-regulated phase may be preferred is in cells where the controlled variable is also used for signaling purposes. For instance, in adipocytes where H_2_O_2_ is used to mediate intracellular insulin signaling, it would be less desirable for an insulin-induced H_2_O_2_ signal to frequently trigger anti-oxidant gene expression, which will otherwise dampen H_2_O_2_ as a second messenger [71].

The second phase of the Y versus S dose response curve is superlinear in appearance and characterized with the highest loop gain. In this phase the homeostatic mechanism operates at full capacity so that cells are best able to effectively resist external disturbance. With a large loop gain, the controlled variable could change very little in response to a wide range of stress levels. But ultimately, activation of gene expression would approach saturation, and the superlinear controlled phase will transition either into the linear phase or directly into the catastrophic sublinear phase, depending on the degree of enzyme saturation by that point. In the two latter phases, the system loses active controls, and persistent elevation of the controlled variable may lead to cell death. Since the feedback regulation in a homeostatic gene network is transcriptionally mediated, it can take hours or longer for the system to settle at a new steady state, as demonstrated in the electrophilic stress response. Before reaching the steady state, the controlled variable may be at very high levels. If cells cannot tolerate such a short-term spike of the controlled variable internally, programs such as apoptosis may be initiated, and the steady-state response for high doses would not be achieved.

The homeostatic benefit of a high loop gain is obvious—it increases the resistance of the cell to external perturbations at low doses, and extends the resistance to higher doses by delaying catastrophic rises in the levels of controlled variables. A high loop gain can be obtained either by concentrating it in one or two local steps or by distributing it more evenly throughout the feedback loop. Overly concentrated loop gains may be less preferred since they may impose special biochemical or energy challenges to the specific reactions/interactions involved. Even if allocating the loop gain more evenly is a better design, some locations within the feedback loop may be preferred over others for gain placement. Among three of the ultrasensitive steps discussed here where local gains can be enhanced—transcription factor multimerization, cooperative binding of transcription factor to response elements, and enzyme multimerization—the latter is probably preferred over the former two when a choice has to be made about gain placement. Although a high loop gain achieved at the two pre-transcriptional locations can definitely enhance the local superlinearity of the controlled phase, thus boosting resistance to perturbations by relatively low-dose stressors, this increased resistance eventually has to succumb to the limiting effect of gene saturation, with the response converging invariably into a similar linear and eventually catastrophic phase (Figure S4A). In contrast, a loop gain enhanced post-transcriptionally through enzyme multimerization cannot only superlinearize the controlled phase further but also extend it, delaying the arrival of the uncontrolled linear and particularly the catastrophic phase (Figure S4B). Since metabolic enzymes usually exist in much higher abundance than transcription factors, the homeostatic benefit of post-transcriptional gain enhancement is nonetheless at the cost of higher energy consumption for synthesizing more enzyme molecules. Additional considerations for gain placement may include avoidance of persistent oscillation, which has been observed in gene regulatory networks with delayed negative feedback [72,73].

Although the present study is concerned with the steady-state behaviors of anti-stress gene regulatory networks, it is important to note that gene expression is intrinsically stochastic and may fluctuate to a great extent around the steady state in both simple and complex gene networks [74–86]. Given the homeostatic objective of an anti-stress gene regulatory network, it is important to understand how cells can cope with this noisy nature of gene expression that may undermine the stability of the intracellular environment. A recent genome-wide study indicated that compared with other genes, those essential to the fitness of organisms are expressed at higher transcription but lower translation rates [87]. This is a strategy believed to lower protein expression noise [74]. Given the fact that most anti-stress genes are indispensable for normal cell functions (the deletion of which is often embryonically lethal or results in severely impaired viability [55,88,89]), a similar expression strategy, i.e., high transcription low translation, may have been adopted by genes responsible for cellular homeostasis to reduce fluctuation. Moreover, the negative feedback nature of anti-stress gene regulatory networks may also help alleviate noise in gene expression. Despite a few studies suggesting that negative autoregulation may increase protein expression noise under certain conditions [90–92], the majority of the literature, including experimental evidence, regards negative feedback as a design that effectively attenuates intrinsic noise in gene expression [6,11,74,90,91,93–98]. Additionally, it has also been shown that dimerization of transcription factors can further reduce protein expression noise in gene feedback loops [93,96]. This noise-reducing interaction resonates with the fact that transcription factors involved in anti-stress regulation often dimerize or trimerize to become active [25,26]. Recently, El-Samad and Khammash suggested that regulated degradation of heat shock factor σ^32^ is a mechanism for suppressing stochastic fluctuation in the heat shock gene regulatory network [98]. In this regard, it is worth mentioning that Nrf2 and HIF, the key transcription factors mediating electrophilic and hypoxic stress responses, respectively, are also regulated primarily through protein degradation [34,99,100]. Taken together, it is highly likely that fluctuations in protein expression and thus controlled variables in anti-stress gene regulatory networks may well be at a minimum through, at least, the above noise-attenuating mechanisms.

A critically important issue in toxicological research and risk assessment is how to estimate low-dose effect from experimental data obtained for high doses. Although linear extrapolation from the high-dose region to the basal point has been a popular practice, in many situations the assumption that dose response relationships in the low-dose region behave linearly does not have theoretical basis. The present study revealed that for cells capable of anti-stress homeostatic regulation, the low-dose region has various nonlinear characteristics. The nature of negative feedback regulation determines that the low-dose region for the controlled variable is basically superlinear, and the stronger the feedback is, the more superlinear it becomes. However, in the presence of diminishing gains for gene activation owing to saturation, the superlinear phase gradually reverses its curvature to become sublinear during its course to join the subsequent linear phase. Therefore, the primary curvature in the low-dose region depends, by and large, on the relative influence from the superlinear controlled phase and the sublinear segment that immediately follows. In conditions where the effect of constitutive activation is insignificant, pre-transcriptional gain is high, and saturation of gene activation occurs early, the superlinear controlled phase appears only transiently, leaving the low-dose region dominated by the sublinear segment. In the presence of feedforward gene activation, the sublinear appearance is even more prominent, and eventually a J-shaped curve could arise. It has long been hypothesized that low-dose effects including hormesis are of homeostatic and adaptive nature [101,102]; our results are consistent with such a concept. Given the diversity and complexity of the dose response curve in the low-dose region, it thus appears inappropriate to extrapolate from high- to low-dose regions with any simple function. Regardless of the curvature within the low-dose region, if linear extrapolation starts from high-dose points in the catastrophic sublinear phase, the low-dose effect is likely to be consistently overestimated, albeit to various degrees. However, if the extrapolation starts from the linear phase or even below, the cellular impact from low doses would be either underestimated or overestimated, depending on the curvature in the low-dose region (Figure 9). For government regulatory purposes, different curvatures in the low-dose region, relative to linear extrapolation, may give rise to significantly different cutoff “safe” exposure levels for a biological stressor of interest. As a result, the difference in the economic cost associated with preventive measures taken to keep exposures below the regulated safe level can be significant.

The present study represents our initial effort to achieve a quantitative understanding of the adaptive cellular response for homeostasis. We realize that the generalized control scheme we studied is a simplification of realistic biological networks that are more complex. But as we showed for the anti-electrophilic defense system, complex feedback networks involving multiple genes, enzymes, and biochemical reactions may observe similar transitions in their controllability. Although the dose response transition we proposed remains to be extensively examined, experimental studies by others on the formation of DNA adducts and protein conjugates [30,31] have provided preliminary evidence indicating the proposed transition may indeed operate in realistic biological systems (Text S5). Clearly, low-dose extrapolations for risk assessment need to acknowledge the complexity of adaptive responses in order to be consistent with biological signaling dynamics and the inherent ability of organisms to detect environmental stressors and maintain homeostatic functions.

## Materials and Methods

Details of the model structures, reactions, and parameter values are provided under Supporting Information, which contains references and rationale for the choice of parameter values. For the generalized control scheme, the parameter values were kept dimensionless and chosen to assist in visualizing the analytical results (Tables S1–S3). For the anti-electrophilic stress model, the parameter values were obtained from the literature if available, or estimated to meet the constraints imposed by experimental observations of our own or others (see Tables S4 and S5 for details). All models were first constructed and parameterized in PathwayLab (InNetics, http://www.innetics.com) and then exported into MatLab (MathWorks, http://www.mathworks.com). All the dose response simulation results were obtained by running the models to steady state in MatLab. Models in the format of MatLab are available for downloading in File Collection S1.

## Supporting Information

Figure S1 Response Coefficient (Gain) and Shape of Dose Response Curve (98 KB PDF)

Figure S2 Model Structure of the Generalized Control Scheme (911 KB PDF)

Figure S3 Simulation Results for the Anti-Electrophilic Gene Regulatory Network Model in Which X Conjugates with GSH Non-Enzymatically (89 KB PDF)

Figure S4 Differential Effect of Varying Pre-Transcriptional versus Post-Transcriptional Gain on Dose Response Curves (78 KB PDF)

File Collection S1 Models in MatLab Format (7 KB ZIP)

Table S1 Reactions, Reaction Rates, and Parameter Values for the Model in Figure S2
(83 KB PDF)

Table S2 Biomolecular Species, ODEs, and Initial Values for the Model in Figure S2
(80 KB PDF)

Table S3 Parameter Values for Simulations Presented in the Main Text and Figure S4
(85 KB PDF)

Table S4 Reaction Rates and Parameter Values for the Anti-Electrophilic Gene Regulatory Network Model (164 KB PDF)

Table S5 Species, ODEs, and Initial Values for the Anti-Electrophilic Gene Regulatory Network Model (118 KB PDF)

Text S1 Equation Derivation (81 KB PDF)

Text S2 Feedforward Gene Activation (97 KB PDF)

Text S3 Biological Examples of Anti-Stress Dose Response Transition (82 KB PDF)

Text S4 Model of Anti-Electrophilic Gene Regulatory Network (58 KB PDF)

Text S5 Negative Feedback with Multiple Genes (96 KB PDF)

## Abbreviations

BST: biochemical systems theory
GCL: glutamate cysteine ligase
GCLC: glutamate cysteine ligase catalytic subunit
GCLM: glutamate cysteine ligase modifier subunit
GS: glutathione synthetase
GSH: glutathione
GST: glutathione S-transferase
GSX: glutathione conjugates
HSF: heat shock transcription factor
Keap1: Kelch-like ECH-associating protein 1
MAPK: mitogen-activated protein kinase
MCA: metabolic control analysis
MRP: multidrug resistance-associated protein
Nrf2: nuclear factor erythroid 2-related factor 2
ROS: reactive oxygen species
