# Supplementary material for: Dose Response Relationship in Anti-Stress Gene Regulatory Networks
Source: PLoS Comput Biol. 2007 Mar 2;3(3):e24. doi: 10.1371/journal.pcbi.0030024 (PMC1808489; doi:10.1371/journal.pcbi.0030024)
Supplement: Supplementary file 1 [file pcbi.0030024.sd001.pdf]

Text S1. Equation derivation

Given the local gains illustrated in Fig. 2A in the main text, and according to signal transfer analysis [1],

$$d\ln Y = r_0 d\ln S + r_3 d\ln G \ , \qquad \text{(S1)}$$

$$d\ln G = r_2 d\ln T \ , \qquad \text{(S2)}$$

$$d\ln T = r_1 d\ln Y \ . \qquad \text{(S3)}$$

Replacing $d\ln G$ in Equation S1 by S2 and S3 sequentially,

$$d\ln Y = r_0 d\ln S + r_1 r_2 r_3 d\ln Y \qquad \text{(S4)}$$

can be arrived, which gives rise to Equation 4 in the main text. Equation 5 and 6 can be similarly derived.

References

1. Kholodenko BN, Hoek JB, Westerhoff HV, Brown GC (1997) Quantification of information transfer via cellular signal transduction pathways. FEBS Lett 414: 430-434.
