# Supplementary material for: Dose Response Relationship in Anti-Stress Gene Regulatory Networks
Source: PLoS Comput Biol. 2007 Mar 2;3(3):e24. doi: 10.1371/journal.pcbi.0030024 (PMC1808489; doi:10.1371/journal.pcbi.0030024)
Supplement: Supplementary file 2 [file pcbi.0030024.sd002.pdf]

Text S2. Feedforward gene activation

In addition to responding to deviations in the controlled variables to activate anti-stress gene expression in a feedback manner, cells may be able to directly sense the presence of the stressor and activate gene expression in a feedforward manner. By driving up anti-stress gene expression independently of the status of the controlled variable, cells may be able to preemptively resist the perturbation by launching a faster response [1]. For instance, in *E. coli* heat shock response, transcription factor σ^{32}, which is responsible for up-regulating heat shock protein expression, can be directly activated by heat through enhanced translation [2]. The systems-level gains for an anti-stress gene network with feedforward activation are as follows:

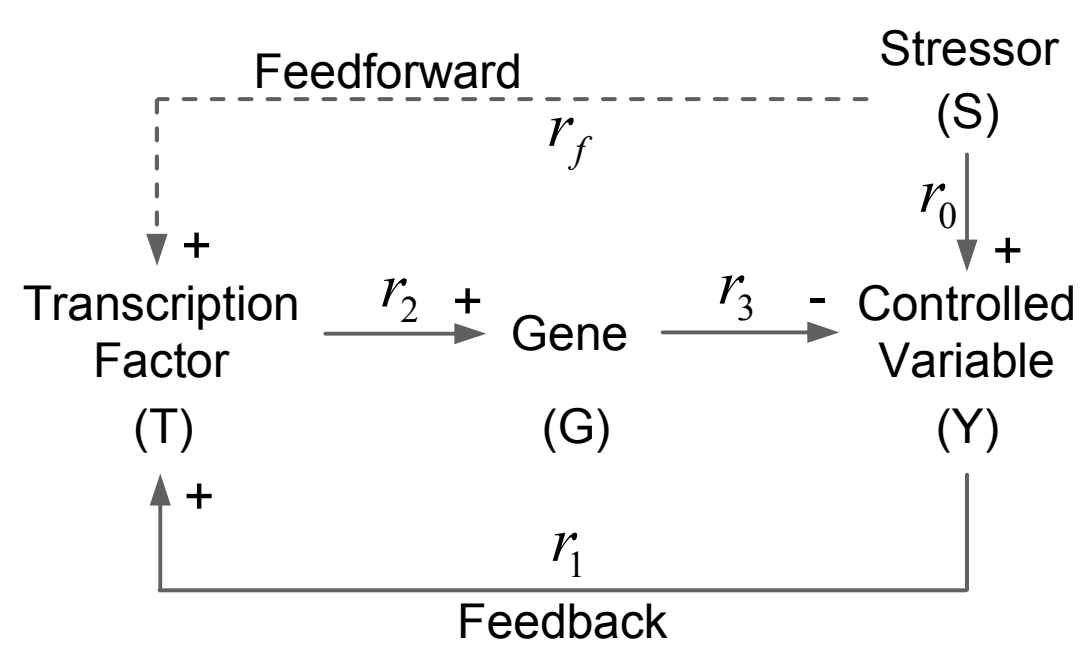

$$R_S^Y = \frac{r_0 - |r_f r_2 r_3|}{1 + |r_1 r_2 r_3|}, \qquad \text{(S5)}$$

$$R_S^T = \frac{r_0 r_1 + r_f}{1 + |r_1 r_2 r_3|}, \qquad \text{(S6)}$$

$$R_S^G = \frac{r_0 r_1 r_2 + r_f r_2}{1 + |r_1 r_2 r_3|}. \qquad \text{(S7)}$$

The term $|r_f r_2 r_3|$ in Equation S5 works to lower the systems-level gain for Y, indicating feedforward activation can increase resistance to perturbation by stressors. Notably, if $|r_f r_2 r_3| > r_0$, then R_S^Y becomes negative, causing depression of Y and ultimately a J-shaped dose response curve.

References

1. El-Samad H, Kurata H, Doyle JC, Gross CA, Khammash M (2005) Surviving heat shock: control strategies for robustness and performance. Proc Natl Acad Sci U S A 102: 2736-2741.
2. Morita M, Kanemori M, Yanagi H, Yura T (1999) Heat-induced synthesis of sigma32 in Escherichia coli: structural and functional dissection of rpoH mRNA secondary structure. J Bacteriol 181: 401-410.
