# Supplementary material for: Dose Response Relationship in Anti-Stress Gene Regulatory Networks
Source: PLoS Comput Biol. 2007 Mar 2;3(3):e24. doi: 10.1371/journal.pcbi.0030024 (PMC1808489; doi:10.1371/journal.pcbi.0030024)
Supplement: Supplementary file 3 [file pcbi.0030024.sd003.pdf]

Text S3. Biological examples of anti-stress dose response transition

In this section, we presented two biological examples from the literature to corroborate the dose response transition presented in the main text. The first example is concerned with carcinogenic DNA adduct O^6-methylguanine (O^6MG) in liver cells capable of DNA repair, the second is concerned with protein conjugation in electrophilic stress response.

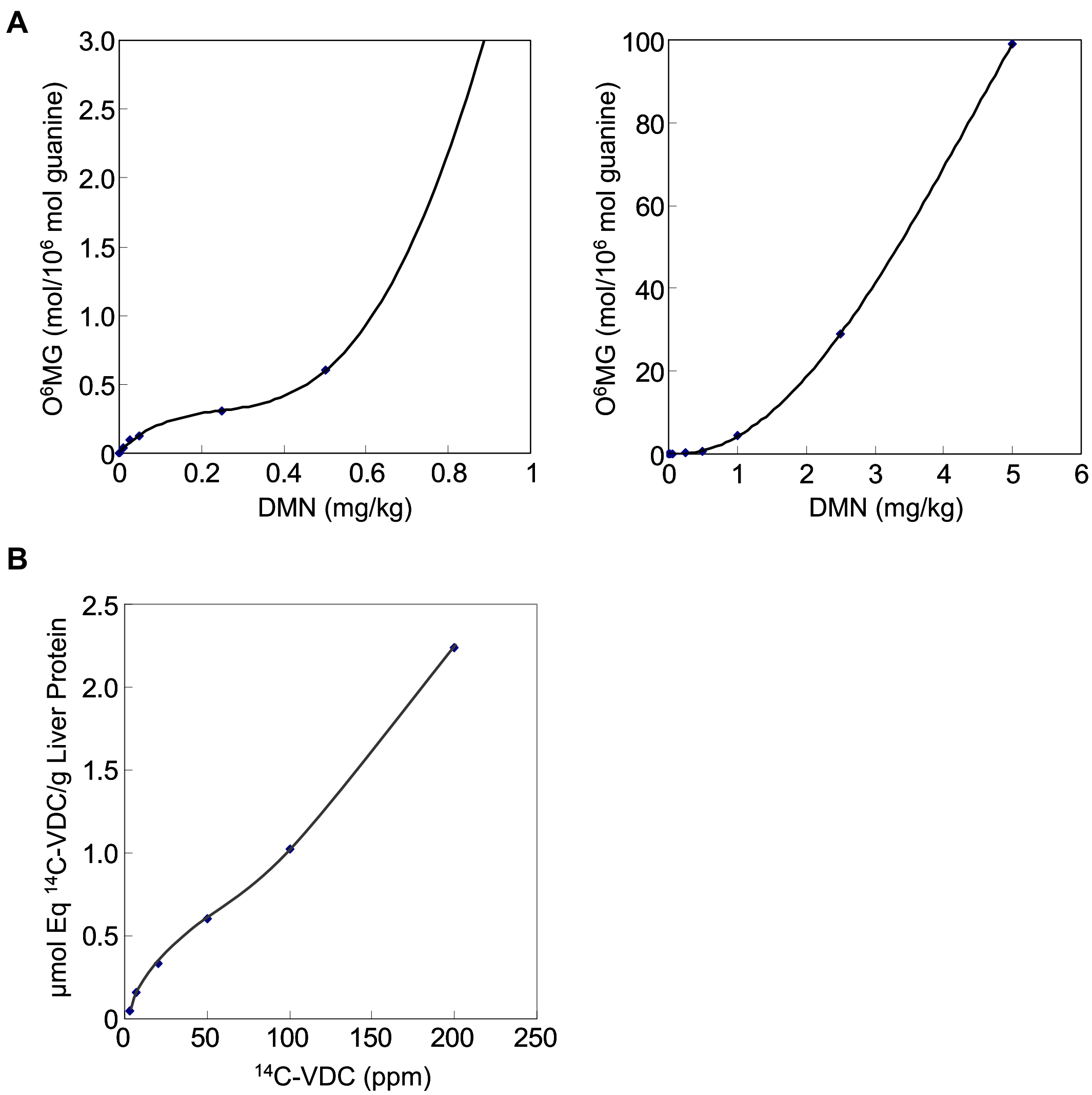

Figure 1. A: Level of DNA adduct O^6-methylguanine (O^6MG) in rat liver cells in response to carcinogen dimethylnitrosamine (DMN). The left panel is a zoomed-in view of the low-dose region in the right panel. At doses greater than 10 mg/kg, the increase in O^6MG tends to be less dramatic (data not shown), reflecting that the total DNA amount is the ultimate limiting factor for the formation of O^6MG in this particular case. Data source: Pegg and Hui, 1978. **B:** Level of liver protein covalently conjugated with VDC metabolite from rats following inhalation exposure to ^{14}C-labled Vinylidene chloride (VDC). Data source: McKenna et al, 1977.

Example #1: DNA repair is an innate defense program protecting genome integrity, and is activated in response to many carcinogenic agents. Carcinogen dimethylnitrosamine (DMN) is believed to exert its cancer-inducing effect through methylating DNA to form DNA adduct O^6MG that is likely to miscode [1,2]. A major DNA repair enzyme responsible for removing O^6MG is methyltransferase which is induced under carcinogenic stress in bacteria and mammalian cells [3,4]. In an early study Pegg and Hui measured the amount of O^6MG in livers from rats exposed to DMN [5]. The dose response relationship between O^6MG and DMN reveals a multi-phasic pattern. It transits from an initial superlinear phase at low doses to a fast-rising sublinear phase at high doses (Fig. 1A, left panel). This transition conforms to the dose response trend we proposed for controlled variables in the main text, and suggests that the control mechanism that regulates the level of DNA adduct O^6MG in liver cells is at full capacity under low doses of DMN, and overwhelmed at higher doses. Notice that the transition profile shown in the left panel is buried and thus visually lost when data at dose higher than 1 mg/kg are plotted together (Fig. 1A, right panel).

Example # 2: Vinylidene chloride (VDC) is an extensively used chemical in the production of plastics. In the liver, it is metabolized into an electrophilic intermediate that is detoxified by conjugation with glutathiol (GSH) [6]. The reactive intermediate can alkylate macromolecules such as proteins to form protein conjugates, causing cytotoxicity [7]. As an electrophile, the intermediate may induce the electrophilic stress response by upregulating the expression of a suite of anti-electrophilic genes as detailed in the corresponding model in the main text. In a study McKenna et al measured the amount of liver protein covalently conjugated with VDC metabolite in rat livers [8]. In the dose range investigated, the relationship between protein conjugate and VDC, when replotted on a linear scale from the original semi-log scale, has a profile that is initially superlinear and then transits through a sublinear segment into a seemingly linear phase (Fig. 1B). This dose response profile is in keeping with the proposed transition preceding the catastrophic phase in the main text.

References

1. Swann PF (1990) Why do O6-alkylguanine and O4-alkylthymine miscode? The relationship between the structure of DNA containing O6-alkylguanine and O4-alkylthymine and the mutagenic properties of these bases. Mutat Res 233: 81-94.
2. Pegg AE (1977) Alkylation of rat liver DNA by dimethylnitrosamine: effect of dosage on O6-methylguanine levels. J Natl Cancer Inst 58: 681-687.
3. Pegg AE (2000) Repair of O(6)-alkylguanine by alkyltransferases. Mutat Res 462: 83-100.
4. Nakabeppu Y, Sekiguchi M (1986) Regulatory mechanisms for induction of synthesis of repair enzymes in response to alkylating agents: ada protein acts as a transcriptional regulator. Proc Natl Acad Sci U S A 83: 6297-6301.
5. Pegg AE, Hui G (1978) Formation and subsequent removal of O6-methylguanine from deoxyribonucleic acid in rat liver and kidney after small doses of dimethylnitrosamine. Biochem J 173: 739-748.
6. Liebler DC, Meredith MJ, Guengerich FP (1985) Formation of glutathione conjugates by reactive metabolites of vinylidene chloride in microsomes and isolated hepatocytes. Cancer Res 45: 186-193.

7. Henschler D, Bonse G (1977) Metabolic activation of chlorinated ethylenes: dependence of mutagenic effect on electrophilic reactivity of the metabolically formed epoxides. Arch Toxicol 39: 7-12.
8. McKenna MJ, Watanabe PG, Gehring PJ (1977) Pharmacokinetics of vinylidene chloride in the rat. Environ Health Perspect 21: 99-105.
