# Supplementary material for: Dose Response Relationship in Anti-Stress Gene Regulatory Networks
Source: PLoS Comput Biol. 2007 Mar 2;3(3):e24. doi: 10.1371/journal.pcbi.0030024 (PMC1808489; doi:10.1371/journal.pcbi.0030024)
Supplement: Supplementary file 4 [file pcbi.0030024.sd004.pdf]

Text S4. Model of anti-electrophilic gene regulatory network

For general description of the mammalian anti-electrophilic stress system, refer to Fig. 10 and the Results section in the main text. Reaction details and parameters are listed in Table S4 and S5. The process of gene activation was modeled as previously [1]. Briefly, the gene can transit between two transcriptional state, i.e., active (on) and inactive (off), and can transcribe only in the active state. In addition to constitutive activation, the transition from the inactive to active state is controlled by Nrf2-Maf bound to the EpREs (For details, see Table S4). Reverting from the active to inactive state is independent of Nrf2-Maf. A cytosolic volume (V_c) of 1000μm^3 and a nuclear volume (V_n) of 100 μm^3 were used.

References

1. Zhang Q, Andersen ME, Conolly RB (2006) Binary Gene Induction and Protein Expression in Individual Cells. Theor Biol Med Model 3: 18.
