# Supplementary material for: Dose Response Relationship in Anti-Stress Gene Regulatory Networks
Source: PLoS Comput Biol. 2007 Mar 2;3(3):e24. doi: 10.1371/journal.pcbi.0030024 (PMC1808489; doi:10.1371/journal.pcbi.0030024)
Supplement: Supplementary file 5 [file pcbi.0030024.sd005.pdf]

Text S5. Negative feedback with multiple genes

In response to cellular stresses, the homeostatic gene regulatory network often drives the expression of more than one genes, which function collectively to counteract the perturbation and maintain the controlled variable within certain levels. For instance, in mammalian electrophilic stress response, there are a suite of genes including *Gclc*, *Gs*, *Gst*, et al, that are up-regulated. For a two-gene system as below, the systems-level gains for Y , T, and each of the two genes are as follows:

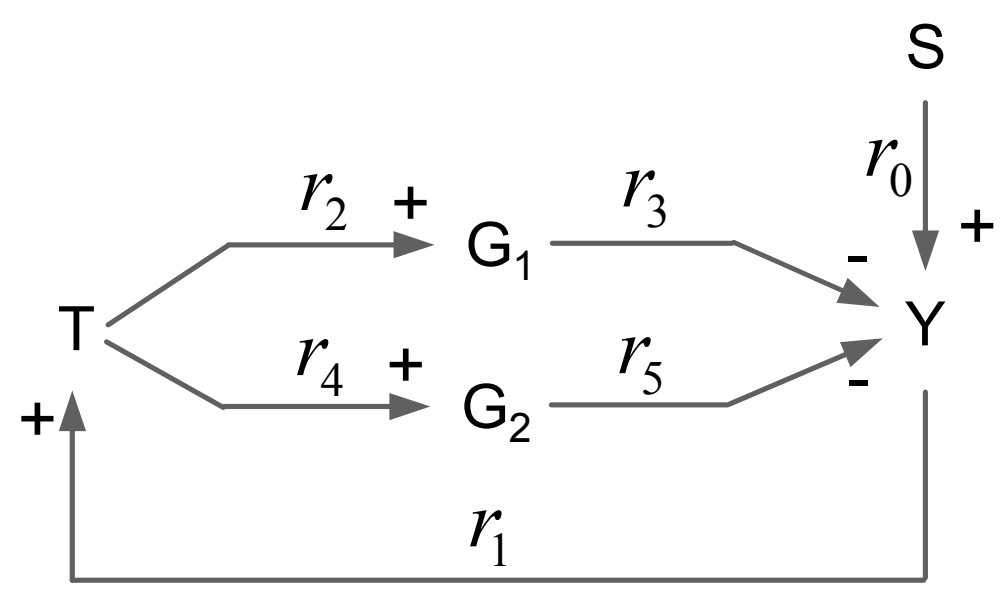

$$R_S^Y = \frac{r_0}{1+\left|r_1(r_2 r_3 + r_4 r_5)\right|}, \quad \text{(S8)}$$

$$R_S^T = \frac{r_0 r_1}{1+\left|r_1(r_2 r_3 + r_4 r_5)\right|}, \quad \text{(S9)}$$

$$R_S^{G_1} = \frac{r_0 r_1 r_2}{1+\left|r_1(r_2 r_3 + r_4 r_5)\right|}, \quad \text{(S10)}$$

$$R_S^{G_2} = \frac{r_0 r_1 r_4}{1+\left|r_1(r_2 r_3 + r_4 r_5)\right|}. \quad \text{(S11)}$$

Equation S8 indicates that the inclusion of additional gene regulations can enhance the loop gain and thus reduce the systems-level gain for the controlled variable Y. Notably, local gain r_2 and r_4 has opposite influence on $R_S^{G_1}$ and $R_S^{G_2}$. An increase in r_2 augments $R_S^{G_1}$, but diminishes $R_S^{G_2}$. Alternatively, an increase in r_4 augments $R_S^{G_2}$, but diminishes $R_S^{G_1}$. The two genes can be functionally related in several ways: 1) G_1 and G_2 can be the two subunits required to form a functional holo-enzyme catalyzing the removal of Y; 2) while G_1 is the enzyme directly responsible for the removal of Y, G_2 participates in a separate reaction which provides the cofactor or co-substrate for G_1-catalyzed reaction; 3) G_1 and G_2 may be two different enzymes involved in two separate reactions, but both of which directly remove Y (e.g., GPx and CAT all remove H_2O_2, but in two distinct reactions); 4) G_1 and G_2 may be two isoforms of the same enzyme family, which are co-expressed in the same cell. In either of these cases, as long as r_3 and r_5 are not zero, enhancing expression of either one of the two genes, by increasing local gain r_2 or r_4 respectively, would reduce the expression level of the other gene. Systems-level gains for gene regulatory networks with more than two genes and/or more than one transcription factors can be similarly derived.
