# Supplementary material for: Dose Response Relationship in Anti-Stress Gene Regulatory Networks
Source: PLoS Comput Biol. 2007 Mar 2;3(3):e24. doi: 10.1371/journal.pcbi.0030024 (PMC1808489; doi:10.1371/journal.pcbi.0030024)
Supplement: Supplementary file 6 [file pcbi.0030024.sg001.pdf]

Figure S1. Response coefficient (gain) and shape of dose response curve

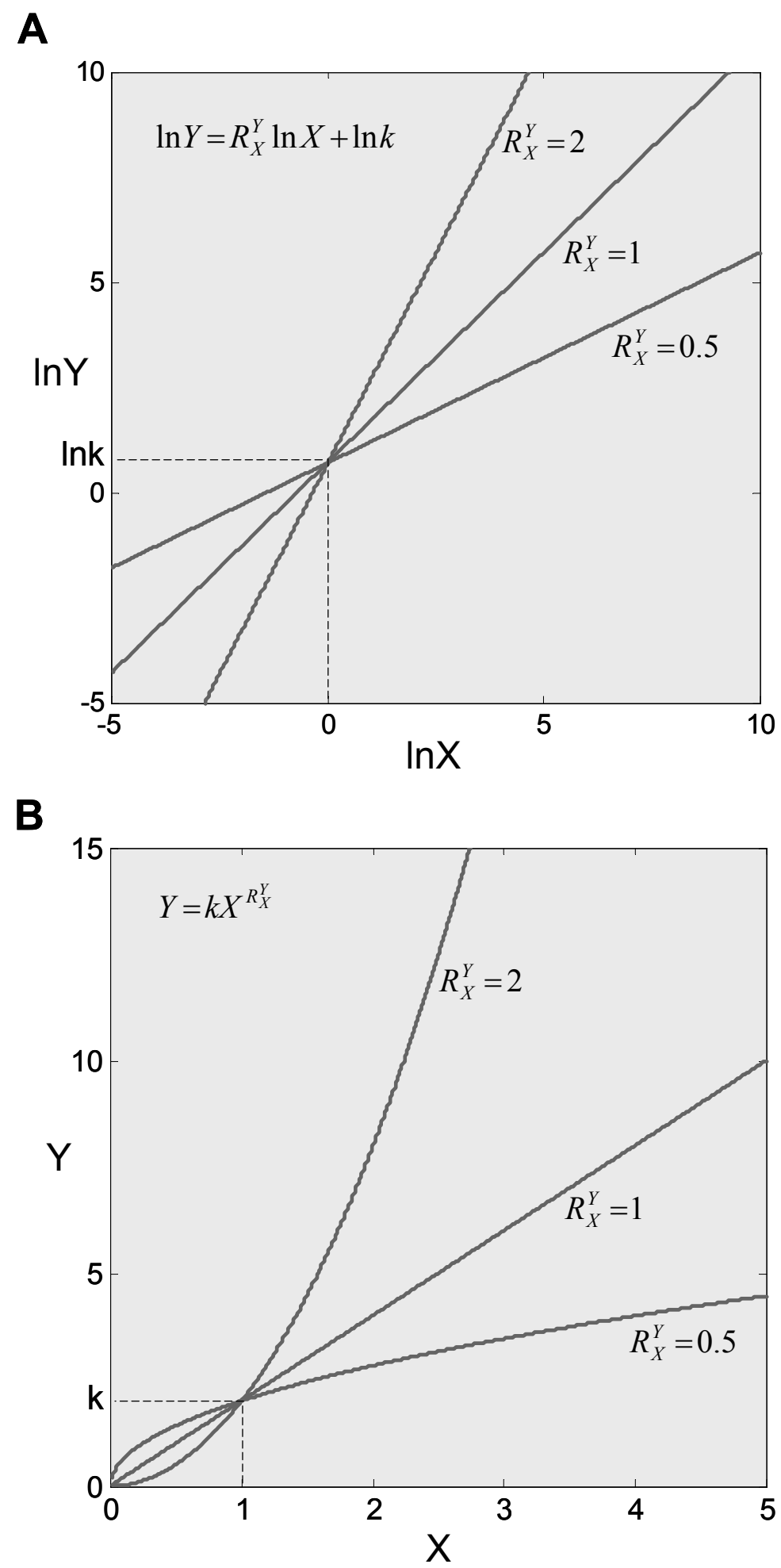

A: On a logarithmic scale, slope of solid lines is indicative of the response coefficient R_X^Y. **B:** On a linear scale, the respective Y vs. S curve is sublinear for $R_X^Y > 1$, linear for $R_X^Y = 1$, and superlinear for $R_X^Y < 1$.
