# Supplementary material for: Dose Response Relationship in Anti-Stress Gene Regulatory Networks
Source: PLoS Comput Biol. 2007 Mar 2;3(3):e24. doi: 10.1371/journal.pcbi.0030024 (PMC1808489; doi:10.1371/journal.pcbi.0030024)
Supplement: Supplementary file 7 [file pcbi.0030024.sg002.pdf]

Figure S2. Model structure of the generalized control scheme

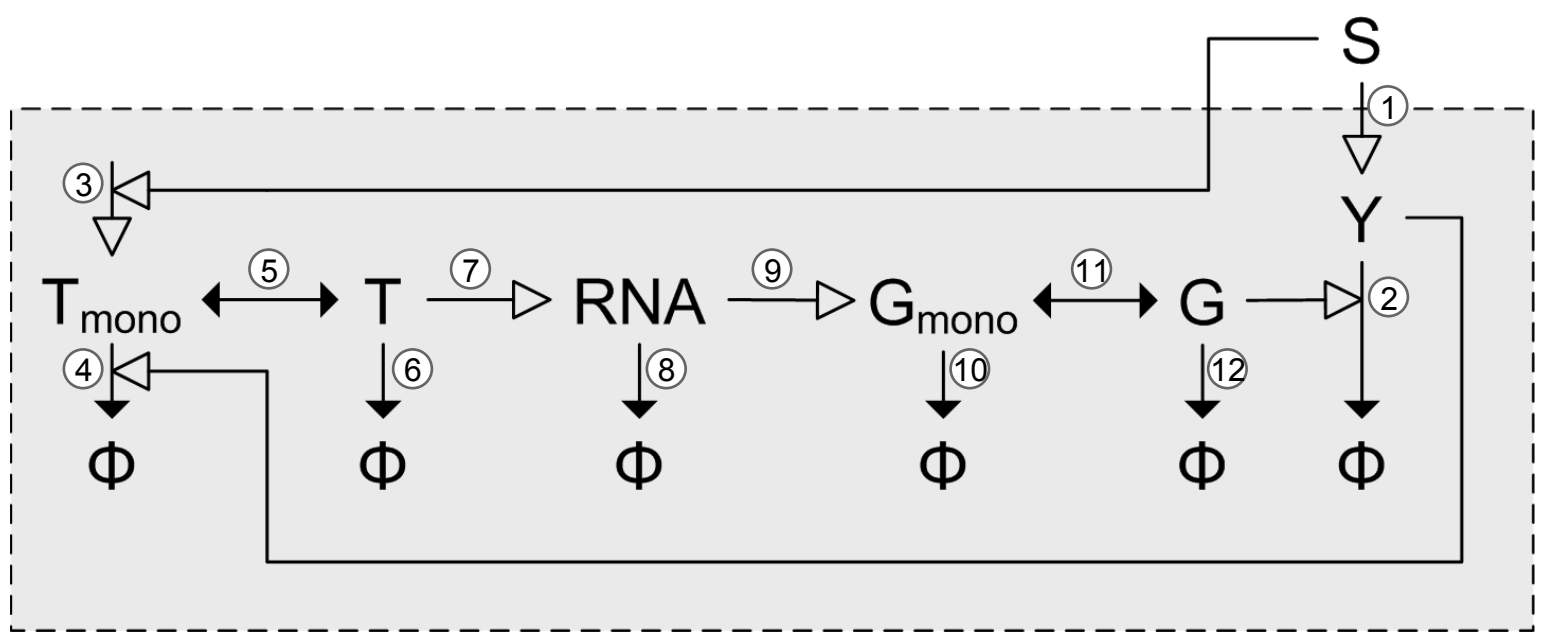

Stressor S increases production of controlled variable Y, which is removed by enzyme G. Y activates transcription factor T_{mono} by decreasing its degradation. T_{mono} can form high-order multimer T, which drives gene transcription. RNA synthesized is translated to G_{mono}, which multimerizes to become fully active enzyme G. In the case of feedforward activation, S directly increases the synthesis of T_{mono}. Solid arrow head: conversion; empty arrow head: activation; Φ: degradation.
