# Supplementary material for: Dose Response Relationship in Anti-Stress Gene Regulatory Networks
Source: PLoS Comput Biol. 2007 Mar 2;3(3):e24. doi: 10.1371/journal.pcbi.0030024 (PMC1808489; doi:10.1371/journal.pcbi.0030024)
Supplement: Supplementary file 8 [file pcbi.0030024.sg003.pdf]

Figure S3. Simulation results for the anti-electrophilic gene regulatory network model in which X conjugates with GSH non-enzymatically.

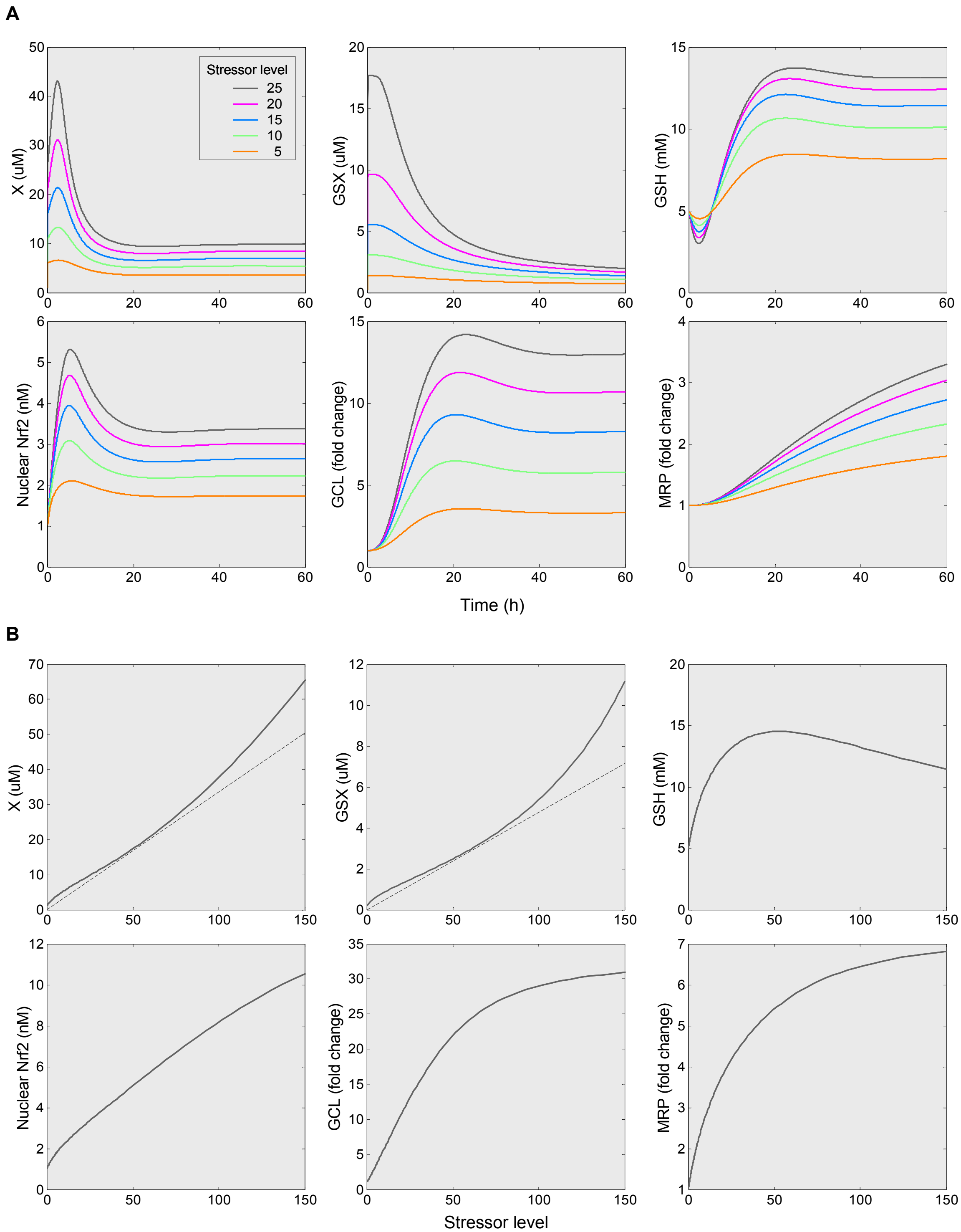

A: Temporal changes in the levels of electrophile X, conjugation product GSX, GSH, Nrf2, GCL, and MRP, in response to different stressor doses. **B:** Steady-state dose response curves for the molecular species listed in (A). Dashed tangent lines originating from (-1, 0) were drawn to help visualizing curvature changes.
