# Supplementary material for: Dose Response Relationship in Anti-Stress Gene Regulatory Networks
Source: PLoS Comput Biol. 2007 Mar 2;3(3):e24. doi: 10.1371/journal.pcbi.0030024 (PMC1808489; doi:10.1371/journal.pcbi.0030024)
Supplement: Supplementary file 9 [file pcbi.0030024.sg004.pdf]

Figure S4. Differential effect of varying pre- vs. post-transcriptional gain on dose response curves

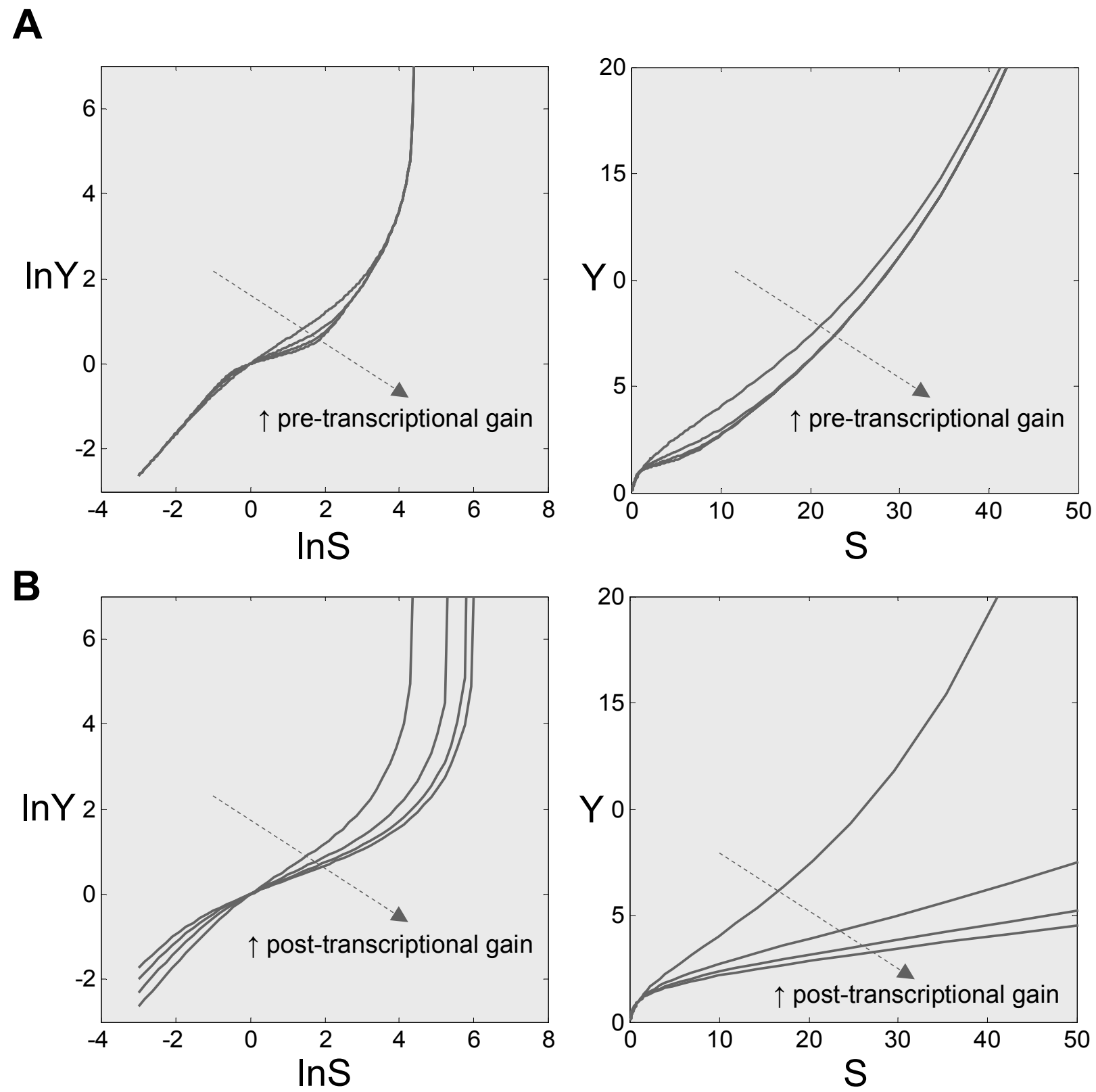

A: With pre-transcriptional amplification Y vs. S dose response curves converge as S increases. Increases in pre-transcriptional gain were modeled by making transcription factor T form increasingly higher-order multimers. **B:** With post-transcriptional amplification Y vs. S dose response curves diverge as S increases. Increases in post-transcriptional gain were modeled by making enzyme G form increasingly higher-order multimers.
