# Supplementary material for: Dose Response Relationship in Anti-Stress Gene Regulatory Networks
Source: PLoS Comput Biol. 2007 Mar 2;3(3):e24. doi: 10.1371/journal.pcbi.0030024 (PMC1808489; doi:10.1371/journal.pcbi.0030024)
Supplement: Supplementary file 10 [file pcbi.0030024.st001.pdf]

Table S1. Reactions, reaction rates, and parameter values for the model in Fig. S2.

Reaction #	Reaction rate	Default parameter value
1	$F_1 = k_1 S$	$k_1 = 10000$
2	$F_2 = k_2 GY$ as linear non-saturable<br>$F_2 = k_{2c} GY/(K_{2m} + Y)$ as saturable	$k_2 = 10$,<br>$k_{2c} = 21000$, $K_{2m} = 2000$
3	$F_3 = k_{3a} + k_{3b} S$	$k_{3a} = 100$, $k_{3b} = 0$
4	$F_4 = k_4 T_{mono}/Y$	$k_4 = 10000$
5	$F_{5f} = k_{5f} T_{mono}^{n_5}$, $F_{5b} = k_{5b} T$	$k_{5f} = 100$, $k_{5b} = 100$, $n_5 = 1$
6	$F_6 = k_6 T$	$k_6 = 0$
7	$F_7 = k_7 T^{n_7} + k_{7a}$ as non-saturable<br>$F_7 = k_{7c} T^{n_7}/(K_{7d}^{n_7} + T^{n_7}) + k_{7b}$ as saturable	$k_7 = 1$, $k_{7a} = 0$, $n_7 = 1$<br>$k_{7b} = 2$, $k_{7c} = 10$, $K_{7d} = 3$, $n_7 = 2$
8	$F_8 = k_8 RNA$	$k_8 = 1$
9	$F_9 = k_9 RNA$	$k_9 = 20$
10	$F_{10} = k_{10} G_{mono}$	$k_{10} = 2$
11	$F_{11f} = k_{11f} G_{mono}^{n_{11}}$, $F_{11b} = k_{11b} G$	$k_{11f} = 100$, $k_{11b} = 100$, $n_{11} = 1$
12	$F_{12} = k_{12} G$	$k_{12} = 0$
