# Supplementary material for: Dose Response Relationship in Anti-Stress Gene Regulatory Networks
Source: PLoS Comput Biol. 2007 Mar 2;3(3):e24. doi: 10.1371/journal.pcbi.0030024 (PMC1808489; doi:10.1371/journal.pcbi.0030024)
Supplement: Supplementary file 11 [file pcbi.0030024.st002.pdf]

Table S2. Biomolecular species, ODEs, and initial values for the model in Fig. S2.

Species	ODE	Initial value
S	$\frac{dS}{dt}=0$	Varied
Y	$\frac{dY}{dt}=F_1-F_2$	100
T_{mono}	$\frac{dT_{mono}}{dt}=F_3-F_4-n_5F_{5f}+n_5F_{5b}$	1
T	$\frac{dT}{dt}=F_{5f}-F_{5b}-F_6$	1
RNA	$\frac{dRNA}{dt}=F_7-F_8$	1
G_{mono}	$\frac{dG_{mono}}{dt}=F_9-F_{10}-n_{11}F_{11f}+n_{11}F_{11b}$	10
G	$\frac{dG}{dt}=F_{11f}-F_{11b}-F_{12}$	10

Note: Simulation results are not affected by initial concentrations.
