# Supplementary material for: Dose Response Relationship in Anti-Stress Gene Regulatory Networks
Source: PLoS Comput Biol. 2007 Mar 2;3(3):e24. doi: 10.1371/journal.pcbi.0030024 (PMC1808489; doi:10.1371/journal.pcbi.0030024)
Supplement: Supplementary file 12 [file pcbi.0030024.st003.pdf]

**Table S3.** Parameter values for simulations presented in the main text and Fig. S4.

| Figure # | Parameter value |
|---|---|
| 3 | A: $k_2 = 10$, $k_7 = 1$, $k_{7a} = 0$, $n_7 = 1$, $n_5 = 1$ (grey), 2 (blue), 3 (green).<br>B: $k_2 = 10$, $k_7 = 1$, $k_{7a} = 0$, blue: $n_7 = 1.5$, green: $k_{11f} = 0.1$, $n_{11} = 4$.<br>C: $k_2 = 10$, $k_7 = 1$, $k_{7a} = 0$, blue: $n_5 = 2$, $n_7 = 1.5$, $k_{11f} = 10$, $n_{11} = 2$, green: $n_5 = 2$, $k_{11f} = 0.1$, $n_{11} = 4$. |
| 4 | $k_2 = 10$, $n_5 = 2$, $k_7 = 1$, $k_{7a} = 4$, $n_7 = 1.5$, $k_8 = 5$. |
| 5 | $k_2 = 10$, $n_5 = 3$, $k_{7b} = 0$, $k_{7c} = 2.44$, $K_{7d} = 1.2$, $n_7 = 2$. |
| 6 | $k_{2c} = 5000$, $K_{2m} = 400$, $n_5 = 2$, $k_7 = 1$, $k_{7a} = 0$, $n_7 = 1.5$. |
| 7 | $k_{2c} = 21000$, $K_{2m} = 2000$, $n_5 = 3$, $k_{7b} = 2$, $k_{7c} = 10$, $K_{7d} = 3$, $n_7 = 2$, $k_8 = 3$. |
| 8 | A: $k_{2c} = 21000$, $K_{2m} = 2000$, $n_5 = 3$, $k_{7b} = 2$, $k_{7c} = 50, 26, 10, 2$, $K_{7d} = 7, 5, 3, 1$, $n_7 = 2$, $k_8 = 3$.<br>B: $k_{2c} = 81000, 41000, 21000, 11000$, $K_{2m} = 8000, 4000, 2000, 1000$, $n_5 = 3$, $k_{7b} = 2$, $k_{7c} = 10$, $K_{7d} = 3$, $n_7 = 2$, $k_8 = 3$. |
| 9 | A: $k_2 = 10$, $n_5 = 1$, $k_{7b} = 2.9$, $k_{7c} = 11$, $K_{7d} = 3$, $n_7 = 2$, $k_8 = 4$, $k_{11f} = 10$, $n_{11} = 2$<br>B: $k_2 = 10$, $n_5 = 2$, $k_{7b} = 2.85$, $k_{7c} = 10.166$, $K_{7d} = 2.8$, $n_7 = 2$, $k_8 = 4$, $k_{11f} = 10$, $n_{11} = 2$<br>C: $k_2 = 10$, $n_5 = 3$, $k_{7b} = 0$, $k_{7c} = 13$, $K_{7d} = 1.5$, $n_7 = 2$, $k_8 = 4$, $k_{11f} = 10$, $n_{11} = 2$<br>D: $k_2 = 10$, $k_{3a} = 50$, $k_{3b} = 50$, $n_5 = 2$, $k_{7b} = 2.85$, $k_{7c} = 10.166$, $K_{7d} = 2.8$, $n_7 = 2$, $k_8 = 4$, $k_{11f} = 10$, $n_{11} = 2$ |
| S4 | A: $k_{2c} = 21000$, $K_{2m} = 2000$, $n_5 = 1, 2, 3, 4$, $k_{7b} = 2$, $k_{7c} = 10$, $K_{7d} = 3$, $n_7 = 2$, $k_8 = 3$.<br>B: $k_{2c} = 21000$, $K_{2m} = 2000$, $k_{7b} = 2$, $k_{7c} = 10$, $K_{7d} = 3$, $n_7 = 2$, $k_8 = 3$, $k_{11f} = 111.34, 12.37, 1.374, 0.153$, $n_{11} = 1, 2, 3, 4$, $k_{12} = 0.2$. |

Note: Parameters that are not assigned values here use default values in Table S1.
