# Supplementary material for: Dose Response Relationship in Anti-Stress Gene Regulatory Networks
Source: PLoS Comput Biol. 2007 Mar 2;3(3):e24. doi: 10.1371/journal.pcbi.0030024 (PMC1808489; doi:10.1371/journal.pcbi.0030024)
Supplement: Supplementary file 13 [file pcbi.0030024.st004.pdf]

Table S4. Reaction rates and parameter values for the anti-electrophilic gene regulatory network model.

Reaction #	Reaction rate	Parameter value	Note
1	$f_1 = k_1[\mathrm{Nrf2\_Keap1}]$	$k_1 = 0.014\ \mathrm{s^{-1}}$	
2	$f_2 = k_2[\mathrm{Nrf2\_Keap1_o}]$	$k_2 = 1\times10^{-4}\ \mathrm{s^{-1}}$	
			These two reactions represents Keap1-mediated Nrf2 degradation. The current consensus on activation of Nrf2 is that electrophilic/oxidative stress conjugates or oxidizes Keap1 (represented as $\mathrm{Keap1_o}$ here), rendering it unable to facilitate Nrf2 degradation [1,2]. Studies have shown that under stresses, Nrf2 half-life lengthens from 13~21 min to 100~200 min [3,4]. k_1, k_2, as well as k_{15} were thus chosen so that at the basal condition overall Nrf2 half-life is 15 min, at fully stressed condition, the half-life relaxes to 115 min. k_2 was set the same as k_{15}, i.e., for free Nrf2.
3	$f_3 = k_{3f}[\mathrm{Nrf2}][\mathrm{Keap1}] - k_{3b}[\mathrm{Nrf2\_Keap1}]$	$k_{3f} = 0.002\ \mathrm{nM^{-1}s^{-1}}$, $k_{3b} = 0.02\ \mathrm{s^{-1}}$	
4	$f_4 = k_{4f}[\mathrm{Nrf2}][\mathrm{Keap1_o}] - k_{4b}[\mathrm{Nrf2\_Keap1_o}]$	$k_{4f} = 0.002\ \mathrm{nM^{-1}s^{-1}}$, $k_{4b} = 0.02\ \mathrm{s^{-1}}$	
			These two reactions represents the binding between Keap1 and Nrf2. Several studies have indicated that the binding affinity between Keap1 and Nrf2 does not differ between unstressed and stressed conditions [1,4-6]. Therefore activation of Nrf2 was not due to increased dissociation from Keap1 sequestration as previously thought. In these studies, the measured Kd between keap1 and Nrf2 was 1 nM, 9 nM, or higher. Here we used 10 nM. The absolute values of these parameters were then chosen to make the binding dynamics in the order of second ~ minute.
5	$f_5 = (k_{5i} + k_{5f}[\mathrm{X}])[\mathrm{Keap1}] - k_{5b}[\mathrm{Keap1_o}]$	$k_{5i} = 0.93\ \mathrm{s^{-1}}$, $k_{5f} = 2.6\times10^{-4}\ \mathrm{nM^{-1}s^{-1}}$, $k_{5b} = 0.1\ \mathrm{s^{-1}}$	
6	$f_6 = (k_{6i} + k_{6f}[\mathrm{X}])[\mathrm{Nrf2\_Keap1}] - k_{6b}[\mathrm{Nrf2\_Keap1_o}]$	$k_{6i} = 0.93\ \mathrm{s^{-1}}$, $k_{6f} = 2.6\times10^{-4}\ \mathrm{nM^{-1}s^{-1}}$, $k_{6b} = 0.1\ \mathrm{s^{-1}}$	
			These two reactions represents Keap1 conjugation/oxidation by electrophile or ROS and its reduction. We assume this occurs with free and Nrf2-bound Keap1 equally. k_{5i} and k_{6i} represents the oxidative activity of species excluding electrophile, such as ROS. Parameter values were chosen so that at the basal condition, electrophile is only responsible for 22% of the oxidative activity. Reversion from $\mathrm{Keap1_o}$ to keap1 follows a constant rate k_{5b} and k_{6b}.
7	$f_7 = k_{7f}[\mathrm{Nrf2}] - k_{7b}[\mathrm{Nrf2_n}]$	$k_{7f} = 2\ \mathrm{\mu m^3 s^{-1}}$, $k_{7b} = 1\ \mathrm{\mu m^3 s^{-1}}$	Translocation of Nrf2 between the cytosol and nucleus. It appears that the nuclear Nrf2 concentration was twice as much as the cytosolic concentration [7], we thus set the ratio between this two parameters to 2. These values were also chosen so that translocation of Nrf2 is not rate-limiting for its nuclear accumulation.
8	$f_8 = k_8[\mathrm{Nrf2_n}]$	$k_8 = 1\times10^{-4}\ \mathrm{s^{-1}}$	Nrf2 degradation in the nucleus. Assume nuclear Nrf2 has a similar half-life as the free Nrf2 in the cytosol.
9	$f_9 = k_{9f}[\mathrm{Nrf2_n}][\mathrm{Maf}] - k_{9b}[\mathrm{Nrf2\_Maf}]$	$k_{9f} = 0.001\ \mathrm{nM^{-1}s^{-1}}$, $k_{9b} = 0.02\ \mathrm{s^{-1}}$	Nrf2 dimerization with Maf. The kinetics are unknown. We set it so that the dissociation dynamics is less than a minute. The current values give a Kd of 20 nM, which is similar to that for estrogen receptor dimerization [8]
10	$f_{10} = k_{10f}[\mathrm{Nrf2\_Maf}]^{N_{10}}[\mathrm{EpRE_{Nrf2}}] - k_{10b}[\mathrm{Nrf2\_Maf\_EpRE_{Nrf2}}]$	$k_{10f} = 0.002\ \mathrm{nM^{-1}s^{-1}}$, $k_{10b} = 0.02\ \mathrm{s^{-1}}$	Binding of Nrf2-Maf dimer to EpRE in *Nrf2* gene promoter. These values, along with values for other genes, were chosen so that the residence time of Nrf2-Maf dimer on EpRE is compatible with the rapid exchange (seconds to minutes) observed between a variety of transcription factors and response elements [9-11]. With the current values, the dissociation constant is 10 nM.
11	$f_{11} = (k_{11i} + k_{11f}[\mathrm{Nrf2\_Maf\_EpRE_{Nrf2}}])[\mathrm{Nrf2_{off}}] - k_{11b}[\mathrm{Nrf2_{on}}]$	$k_{11i} = 0.0025\ \mathrm{s^{-1}}$, $k_{11f} = 100\ \mathrm{nM^{-1}s^{-1}}$, $k_{11b} = 0.01\ \mathrm{s^{-1}}$	Activation of *Nrf2* gene. These values, along with values for other genes, were chosen so that the dynamics of the adaptive response is not determined by the kinetics at the promoter, but rather by

	the half-lives of mRNAs and proteins of Nrf2 and other anti-electrophilic genes in the model. For Nrf2, the values were also set such that at the basal condition the *Nrf2* gene operates at about half of its capacity. This ensures that even at full induction by oxidative stress, the increase in Nrf2 mRNA level can not exceed 2 fold, as observed experimentally with arsenic exposure [7,12].	
12	$f_{12} = k_{12}[Nrf2_{on}]$ Transcription of Nrf2 mRNA. The value was chosen so that basal nuclear Nrf2 concentration is about 1 nM.	$k_{12} = 2.04\times10^{-3}$ s^{-1}
13	$f_{13} = k_{13}[Nrf2mRNA]$ Nrf2 mRNA degradation in the cytosol. The value reflects a half-life of 3 h observed in HepG2 cells [13].	$k_{13} = 6.43\times10^{-5}$ s^{-1}
14	$f_{14} = k_{14}[Nrf2mRNA]$ Translation of Nrf2. This value gives about 150 protein molecules produced per mRNA per hour, an average found in eukaryotic cells [14,15]. Translation rates for protein GCLC (k_{20}), GCLM (k_{26}), GS (k_{34}), GST (k_{42}), and MRP (k_{50}) used the same value.	$k_{13} = 0.0417$ s^{-1}
15	$f_{15} = k_{15}[Nrf2]$ Free Nrf2 degradation in the cytosol. This value gives a half-life of 115 min, which is in the range of 100~200 min as measured experimentally [3,4].	$k_{15} = 1\times10^{-4}$ s^{-1}
16	$f_{16} = k_{16f}[Nrf2\_Maf]^{N_{16}}[EpRE_{GCLC}] - k_{16b}[Nrf2\_Maf\_EpRE_{GCLC}]$ Binding of Nrf2-Maf dimer to EpRE in *Gclc* gene promoter. Cooperative binding was implemented here to enhance *Gclc* expression. The values were chosen so that the time-course profiles of both Nrf2 and GCLC can simultaneously and quantitatively match the respective experimental observations in RAW macrophages treated with hypochlorous acid (unpublished data, Pi et al.).	$k_{16f} = 0.8$ nM^{-3}s^{-1}, $k_{16b} = 0.02$ s^{-1}, $N_{16} = 3$
17	$f_{17} = (k_{17i} + k_{17f}[Nrf2\_Maf\_EpRE_{GCLC}])[GCLC_{off}] - k_{17b}[GCLC_{on}]$ Activation of *Gclc* gene. It contains both constitutive and Nrf2-activated components. With these values, steady-state GCLC mRNA level in the model drops to 41% in the absence of *Nrf2* gene, which is in the range of 16%~65% observed experimentally in *Nrf2*(-/-) cells and mice [16-19]. Together with decrease in GCLM, GSH level decreases correspondingly to 58%, which is also compatible with the 50%~71% range observed experimentally in *Nrf2*(-/-) cells and mice [17,18,20]. Moreover, *Nrf2*(-/-) mouse macrophages only show minimal decreases in GSH levels compared with *Nrf2*(+/-) [21]. Consistent to this result, in our model, GSH level only decreases from 3.1 mM in *Nrf2*(+/-) to 2.87 mM in *Nrf2*(-/-). Also see reaction 11 for rationale on the dynamics.	$k_{17i} = 4\times10^{-4}$ s^{-1}, $k_{17f} = 8.7$ nM^{-1}s^{-1}, $k_{17b} = 0.01$ s^{-1}
18	$f_{18} = k_{18}[GCLC_{on}]$ Transcription of GCLC mRNA. The value and that for GCLM mRNA were chosen so that at the basal condition 1) GCL and GCLC are at concentrations maintaining a GSH production rate of close to 400 nM/s as specified in the GCLC and GCL entry in Table S5; 2) the ratio of GCLC/GCL is at 3 as specified in reaction 28.	$k_{18} = 0.66$ s^{-1}
19	$f_{19} = k_{19}[GCLCmRNA]$ GCLC mRNA degradation. The value reflects a half-life of 4 h observed in both HepG2 and L2 cells [22,23].	$k_{19} = 4.83\times10^{-5}$ s^{-1}
20	$f_{20} = k_{20}[GCLCmRNA]$ Translation of GCLC. See reaction 14 for explanation of the parameter value.	$k_{20} = 0.0417$ s^{-1}
21	$f_{21} = k_{21}[GCLC]$ GCLC degradation. The half-life of GCLC is unknown. Here we adjusted the value, together with those for the degradation of the subunit GCLM and holoenzyme GCL, to give a half-life of 5 h. This value, together with that for GCLM degradation, allows the increase in GSH to peak around 12 h, as occurring in RAW macrophages under exposure to hypochlorous acid (unpublished data, Pi et al.).	$k_{21} = 3.86\times10^{-5}$ s^{-1}
22	$f_{22} = k_{22f}[Nrf2\_Maf]^{N_{22}}[EpRE_{GCLM}] - k_{22b}[Nrf2\_Maf\_EpRE_{GCLM}]$	$k_{22f} = 0.8$ nM^{-3}s^{-1}, $k_{22b} = 0.02$ s^{-1}, $N_{22} = 3$

	Binding of Nrf2-Maf dimer to EpRE in *Gclm* gene promoter. Cooperative binding was implemented here to enhance *Gclm* expression. The values were chosen so that the time-course profile of GSH can match quantitatively the experimental observation in RAW macrophages treated with hypochlorous acid (unpublished data, Pi et al.).	
23	$f_{23} = (k_{23i} + k_{23f}[Nrf2\_Maf\_EpRE_{GCLM}])[GCLM_{off}] - k_{23b}[GCLM_{on}]$<br>Activation of *Gclm* gene. It contains both constitutive and Nrf2-activated components. With these values, steady-state GCLM mRNA level in the model drops to 54% in the absence of *Nrf2* gene. Reported decrease in GCLM mRNA levels in *Nrf2*(-/-) cells and mice ranged from 12.5% to no change [17,18]. Together with decrease in GCLC, GSH level decreases correspondingly to 58%, which is also compatible with the 50%~71% range observed experimentally in both *Nrf2*(-/-) cells and mice [17,18,20]. Also see reaction 11 for rationale on the dynamics.	$k_{23i} = 1.3\times10^{-4}$ s^{-1},<br>$k_{23f} = 1.6$ $nM^{-1}s^{-1}$, $k_{11b} = 0.01$ s^{-1}
24	$f_{24} = k_{24}[GCLM_{on}]$<br>Transcription of GCLM mRNA. See reaction 18 for explanation of the parameter value.	$k_{24} = 1.07$ s^{-1}
25	$f_{25} = k_{25}[GCLMmRNA]$<br>GCLM mRNA degradation. Half-life of GCLM mRNA is 3.5 and 8 h in L2 and HepG2 cells, respectively [22,23]. The value used here gives a half-life of 4 h.	$k_{25} = 4.83\times10^{-5}$ s^{-1}
26	$f_{26} = k_{26}[GCLMmRNA]$<br>Translation of GCLM. See reaction 14 for explanation of the parameter value.	$k_{26} = 0.0417$ s^{-1}
27	$f_{27} = k_{27}[GCLM]$<br>GCLM degradation. The half-life of GCLM is unknown. See reaction 21 for explanation of the parameter value.	$k_{27} = 3.86\times10^{-5}$ s^{-1}
28	$f_{28} = k_{28f}[GCLC][GCLM] - k_{28b}[GCL]$<br>Dimerization of GCLC and GCLM to form the holoenzyme GCL. The parameter values were chosen here to meet two conditions. 1) They give a GCLC/GCL ratio close to 3, which is in the range of 2~7 in various tissues observed by Chen et al [24]. 2) The GSH level decreases to 80%, 72%, 24% for *Gclc*(+/-), *Gclm*(+/-),and *Gclm*(-/-) gene deficiency, respectively. The experimentally observed decreases are 80%, 43%~83%, and 9%~16%, respectively [25,26]. With these values, the dissociation constant is at 1μM.	$k_{28f} = 2\times10^{-5}$ $nM^{-1}s^{-1}$, $k_{28b} = 0.02$ s^{-1}
29	$f_{29} = k_{29}[GCL]$<br>Holoenzyme GCL degradation. The half-life of GCL is unknown. See reaction 21 for explanation of the parameter value.	$k_{29} = 3.86\times10^{-5}$ s^{-1}
30	$f_{30} = k_{30f}[Nrf2\_Maf]^{N_{30}}[EpRE_{GS}] - k_{30b}[Nrf2\_Maf\_EpRE_{GS}]$<br>Binding of Nrf2-Maf dimer to EpRE in *Gs* gene promoter. Cooperative binding was implemented here to enhance *Gs* expression.	$k_{30f} = 0.042$ $nM^{-2}s^{-1}$, $k_{30b} = 0.02$ s^{-1},<br>$N_{30} = 2$
31	$f_{31} = (k_{31i} + k_{31f}[Nrf2\_Maf\_EpRE_{GS}])[GS_{off}] - k_{31b}[GS_{on}]$<br>Activation of *Gs* gene. It contains both constitutive and Nrf2-activated components. With these values, steady-state GS mRNA level in the model drops to 54% in the absence of *Nrf2* gene. Also see reaction 11 for rationale on the dynamics.	$k_{31i} = 5\times10^{-4}$ s^{-1},<br>$k_{31f} = 5.95$ $nM^{-1}s^{-1}$, $k_{31b} = 0.01$ s^{-1}
32	$f_{32} = k_{32}[GS_{on}]$<br>Transcription of GS mRNA. The value was chosen so that at the basal condition GS is at a concentration maintaining a r-GC level of 0.15 mM as specified in the r-GC entry in Table S5.	$k_{32} = 0.38$ s^{-1}
33	$f_{33} = k_{33}[GSmRNA]$<br>GS mRNA degradation. The half-life of GS is unknown, we assumed it is 4 h.	$k_{33} = 4.83\times10^{-5}$ s^{-1}
34	$f_{34} = k_{34}[GSmRNA]$	$k_{34} = 0.0417$ s^{-1}

	Translation of GS monomer. See reaction 14 for explanation of the parameter value.	
35	$f_{35} = k_{35}[GS_{mono}]$<br>GS monomer degradation. The half-life of GS monomer is unknown, we assumed it is 5 h.	$k_{35} = 3.86\times10^{-5}$ s^{-1}
36	$f_{36} = k_{36f}[GS_{mono}]^2 - k_{36b}[GS]$<br>Homodimerization of GS monomers to form dimer GS. The kinetics are unknown. The values used here give a dissociation constant of 0.1 μM.	$k_{36f} = 2\times10^{-4}$ nM^{-1}s^{-1}, $k_{36b} = 0.02$ s^{-1}
37	$f_{37} = k_{37}[GS]$<br>GS degradation. The half-life of GS is unknown. In keeping with the concept that fully active enzymes are generally more stable than their subunits, we assumed the half-life of GS is 10 h, which is twice that of the monomers.	$k_{37} = 1.93\times10^{-5}$ s^{-1}
38	$f_{38} = k_{38f}[Nrf2\_Maf]^{N_{38}}[EpRE_{GST}] - k_{38b}[Nrf2\_Maf\_EpRE_{GST}]$<br>Binding of Nrf2-Maf dimer to EpRE in *Gst* gene promoter. Cooperative binding was implemented here to enhance *Gst* expression.	$k_{38f} = 0.042$ nM^{-2}s^{-1}, $k_{38b} = 0.02$ s^{-1}, $N_{38} = 2$
39	$f_{39} = (k_{39i} + k_{39f}[Nrf2\_Maf\_EpRE_{GST}])[GST_{off}] - k_{39b}[GST_{on}]$<br>Activation of *Gst* gene. It contains both constitutive and Nrf2-activated components. With these values, steady-state GST mRNA level in the model drops to 54% in the absence of *Nrf2* gene, which is compatible to the 40%~66% reported range [19,27]. Also see reaction 11 for rationale on the dynamics.	$k_{39i} = 1\times10^{-3}$ s^{-1}, $k_{39f} = 11.9$ nM^{-1}s^{-1}, $k_{39b} = 0.01$ s^{-1}
40	$f_{40} = k_{40}[GST_{on}]$<br>Transcription of GST mRNA. The value was chosen so that at the basal condition GST is at a concentration maintaining a X level of 1 uM as specified in the X entry in Table S5.	$k_{40} = 0.081$ s^{-1}
41	$f_{41} = k_{41}[GSTmRNA]$<br>GST mRNA degradation. The value used here gives a half life of 4.1 h, as observed for GST pi isoform mRNA in human colon cancer cells [28]	$k_{41} = 4.71\times10^{-5}$ s^{-1}
42	$f_{42} = k_{42}[GSTmRNA]$<br>Translation of GST monomer. See reaction 14 for explanation of the parameter value.	$k_{42} = 0.0417$ s^{-1}
43	$f_{43} = k_{43}[GST_{mono}]$<br>GST monomer degradation. GST pi isoform protein half-life is 1~2 h in human colon cancer cells [28]. We used 1.5 h.	$k_{43} = 1.29\times10^{-4}$ s^{-1}
44	$f_{44} = k_{44f}[GST_{mono}]^2 - k_{44b}[GST]$<br>Homodimerization of GST monomers to form dimer GST. The kinetics are unknown. The values used here give a dissociation constant of 0.1 μM.	$k_{44f} = 2\times10^{-4}$ nM^{-1}s^{-1}, $k_{44b} = 0.02$ s^{-1}
45	$f_{45} = k_{45}[GST]$<br>GST degradation. In keeping with the concept that fully active enzymes are generally more stable than their subunits, we assumed the half-life of GST is 15 h.	$k_{45} = 1.29\times10^{-5}$ s^{-1}
46	$f_{46} = k_{46f}[Nrf2\_Maf]^{N_{46}}[EpRE_{MRP}] - k_{46b}[Nrf2\_Maf\_EpRE_{MRP}]$<br>Binding of Nrf2-Maf dimer to EpRE in *Mrp* gene promoter. Several consensus EpREs have been identified in the promoter of *Mrp1* in mice and human [19]. Cooperative binding was implemented here to enhance *Mrp* expression.	$k_{46f} = 0.042$ nM^{-2}s^{-1}, $k_{46b} = 0.02$ s^{-1}, $N_{46} = 2$
47	$f_{47} = (k_{47i} + k_{47f}[Nrf2\_Maf\_EpRE_{MRP}])[MRP_{off}] - k_{47b}[MRP_{on}]$<br>Activation of *Mrp* gene. It contains both constitutive and Nrf2-activated components. With these values, steady-state MRP mRNA and MRP protein levels in the model drops to 39% and 29% respectively in the absence of *Nrf2* gene. This magnitude of decrease approximately matches the	$k_{47i} = 0.68\times10^{-3}$ s^{-1}, $k_{47f} = 16$ nM^{-1}s^{-1}, $k_{47b} = 0.01$ s^{-1}

	38% and 32% drop reported in the fibroblast in *Nrf*2(-/-) mice [19]. Also see reaction 11 for rationale on the dynamics.
48	$f_{48} = k_{48}[MRP_{on}]$ $k_{48} = 8.2$ s^{-1}<br>Transcription of MRP mRNA. The value was chosen so that at the basal condition MRP is at a concentration maintaining a GSX level of 0.2 uM as specified in the GSX entry in Table S5.
49	$f_{49} = k_{49}[MRPmRNA]$ $k_{49} = 1.93\times10^{-5}$ s^{-1}<br>MRP mRNA degradation. The half-life is unknown, we assumed it is 10 h.
50	$f_{50} = k_{50}[MRPmRNA]$ $k_{50} = 0.0417$ s^{-1}<br>Translation of GST monomer. See reaction 14 for explanation of the parameter value.
51	$f_{51} = k_{51}[MRP_{mono}]$ $k_{51} = 1.93\times10^{-5}$ s^{-1}<br>MRP monomer degradation. The half-life of MRP monomer is unknown, we assumed it is 10 h, which is about 1/3 of the dimer.
52	$f_{52} = k_{52f}[MRP_{mono}]^2 - k_{52b}[MRP]$ $k_{52f} = 1\times10^{-5}$ nM^{-1}s^{-1}, $k_{52b} = 0.02$ s^{-1}<br>Homodimerization of MRP monomers to form dimer MRP. The kinetics are unknown. The values used here give a dissociation constant of 2 μM.
53	$f_{53} = k_{53}[MRP]$ $k_{53} = 7.15\times10^{-6}$ s^{-1}<br>MRP degradation. This value gives a half-life of 27 h, as reported for MRP2 in rat liver cells [29].
54	$f_{54} = k_c[GCL]\frac{[ATP]}{K_{m(ATP)}(1+\frac{[GSH]}{K_{is(ATP)}})+[ATP](1+\frac{[GSH]}{K_{ii(ATP)}})}\times\frac{[Glu]}{K_{m(Glu)}(1+\frac{[GSH]}{K_{is(Glu)}})+[Glu](1+\frac{[GSH]}{K_{ii(Glu)}})}\times\frac{[Cys]}{K_{m(Cys)}+[Cys]}$<br>Synthesis of γ-glutamylcysteine by GCL and GCLC. The above equation gives the rate by GCL, GCLC follows the same rate equation with different parameter values. The overall synthesis rate is the sum of that catalyzed by GCL and that by GCLC. According to [24], this reaction is modeled as an irreversible random reaction, and GSH noncompetitively inhibits GCL and GCLC through binding sites for both glutamate (Glu) and ATP. The following parameter values are taken from mouse GCL and GCLC [24].<br>For GCL the parameter values are: $k_c = 8.2$ s^{-1}, $K_{m(ATP)} = 0.87\times10^6$ nM, $K_{is(ATP)} = 6.5\times10^6$ nM, $K_{ii(ATP)} = 3.9\times10^6$ nM, $K_{m(Glu)} = 0.48\times10^6$ nM, $K_{is(Glu)} = 0.8\times10^6$ nM, $K_{ii(Glu)} = 3.1\times10^6$ nM, $K_{m(Cys)} = 0.22\times10^6$ nM.<br>For GCLC the parameter values are: $k_c = 1.9$ s^{-1}, $K_{m(ATP)} = 5\times10^6$ nM, $K_{is(ATP)} = 1.3\times10^6$ nM, $K_{ii(ATP)} = 0.4\times10^6$ nM, $K_{m(Glu)} = 1.6\times10^6$ nM, $K_{is(Glu)} = 0.3\times10^6$ nM, $K_{ii(Glu)} = 0.8\times10^6$ nM, $K_{m(Cys)} = 0.27\times10^6$ nM.
55	$f_{55} = k_c[GS]\frac{\frac{[rGC]}{K_{m1(rGC)}}+\frac{[rGC]^2}{K_{m1(rGC)}K_{m2(rGC)}}}{1+\frac{2[rGC]}{K_{m1(rGC)}}+\frac{[rGC]^2}{K_{m1(rGC)}K_{m2(rGC)}}}\times\frac{[Gly]}{K_{m(Gly)}+[Gly]}\times\frac{[ATP]}{K_{m(ATP)}+[ATP]}$<br>Synthesis of GSH by GS. This reaction is modeled as an irreversible random reaction, with rGC exerting some negative cooperativity as suggested by [30,31]. The following parameter values are taken from human GS [31].<br>$k_c = 6.5$ s^{-1}, $K_{m1(rGC)} = 0.66\times10^6$ nM, $K_{m2(rGC)} = 1.5\times10^6$ nM, $K_{m(Gly)} = 1.75\times10^6$ nM, $K_{m(ATP)} = 0.07\times10^6$ nM
56	$f_{56} = \frac{V_{m56}[GSH]}{K_{m56}+[GSH]}$ $K_{m56} = 20\times10^6$ nM<br>$V_{m56} = 1.845\times10^3$ nM s^{-1}

	This reaction lumps all the GSH consumptions except that through the GST pathway, and is expressed in a simple Michalis-Menten form. This mainly includes GSH efflux and net GSH conversion to GSSG. It appears that GSH efflux is the dominant form of consumption in most of cell types [32-34]. This is also corroborated by the exponential decay of GSH after inhibition of its synthesis [33]. Were GSH primarily consumed through enzymatic reactions including those catalyzed by GST and GPx, the decay profile would have been more linear since GSH concentration is usually far greater than its Km. The reported GSH efflux rate ranges from 200~1000 nM/s in various cell types [32,33,35,36]. In our model the efflux rate is 366 nM/s at the basal condition. These parameter values ensure that basal GSH concentration is about 5mM, which is the middle point of the reported 1-10 mM range [34].
57	For enzymatic conjugation: $f_{57} = k_c[\mathrm{GST}]\dfrac{[\mathrm{GSH}]}{(K_{m(GSH)}(1+\dfrac{[\mathrm{GSX}]}{K_{i(GSH)}})+[\mathrm{GSH}])} \times \dfrac{[\mathrm{X}]}{(K_{m(X)}+[\mathrm{X}])(1+\dfrac{[\mathrm{GSX}]}{K_{i(X)}})}$ Conjugation of electrophile X with GSH by GST. This reaction is modeled as an irreversible sequential random reaction with competitive product inhibition on GSH and noncompetitive inhibition on 4-hydroxynonenal (HNE) [37,38]. The following parameter values are for 4-hydroxnonenal [38], except $K_{m(GSH)}$ for 1-chloro-2,4-dinitrobenzene (CDNB) [39]. $k_c = 7.67$ s^{-1}, $K_{m(GSH)} = 0.5\times10^6$ nM, $K_{i(GSH)} = 0.085\times10^6$ nM, $K_{m(X)} = 0.05\times10^6$ nM, $K_{i(X)} = 0.085\times10^6$ nM. For non-enzymatic conjugation: $f_{57} = k_c[\mathrm{X}][\mathrm{GSH}]$, where $k_c = 4.04\times10^{-9}$ nM^{-1} s^{-1} to keep the basal X level the same as the case of enzymatic reaction.
58	$f_{58} = \dfrac{k_{c58}[\mathrm{MRP}][\mathrm{GSX}]}{K_{m58}+[\mathrm{GSX}]}$ $k_{c58} = 0.1$ s^{-1}, $K_{m58} = 7\times10^3$ nM Export of GS-conjugated X (GSX) by MRP. The value of k_{c58} falls into the middle of the 0.012~13.3 s^{-1} range reported for a variety of chemicals for MRP2 in 4 different species [40]. The value of K_{m58} is also in the range of 0.53 ~ 9.79 uM as reported in [40].
59	$f_{59} = k_{59}$ $k_{59} = 20$ nM s^{-1} This reaction represents the basal endogenous production of electrophile X including HNE. The production rate was chosen to assure that GSH consumption through GST is only a fraction of its total consumption, as the majority of GSH is removed through efflux [32-34]. In this case, the fraction was set to be about 5%.
60	$f_{60} = k_{60}[\mathrm{stressor}]$ $k_{60} = 20$ nM s^{-1} This reaction represents the production of electrophile by external stressors. k_{52} was set equal to k_{51} so that the stressor level can be quantitatively expressed relative to the basal electrophile production rate.

References

1. Kobayashi A, Kang MI, Watai Y, Tong KI, Shibata T, et al. (2006) Oxidative and Electrophilic Stresses Activate Nrf2 through Inhibition of Ubiquitination Activity of Keap1. Mol Cell Biol 26: 221-229.
2. Hong F, Sekhar KR, Freeman ML, Liebler DC (2005) Specific patterns of electrophile adduction trigger Keap1 ubiquitination and Nrf2 activation. J Biol Chem 280: 31768-31775.
3. Stewart D, Killeen E, Naquin R, Alam S, Alam J (2003) Degradation of transcription factor Nrf2 via the ubiquitin-proteasome pathway and stabilization by cadmium. J Biol Chem 278: 2396-2402.
4. He X, Chen MG, Lin GX, Ma Q (2006) Arsenic induces NAD(P)H-quinone oxidoreductase I by disrupting the Nrf2 x Keap1 x Cul3 complex and recruiting Nrf2 x Maf to the antioxidant response element enhancer. J Biol Chem 281: 23620-23631.
5. Eggler AL, Liu G, Pezzuto JM, van Breemen RB, Mesecar AD (2005) Modifying specific cysteines of the electrophile-sensing human Keap1 protein is insufficient to disrupt binding to the Nrf2 domain Neh2. Proc Natl Acad Sci U S A 102: 10070-10075.
6. Tong KI, Katoh Y, Kusunoki H, Itoh K, Tanaka T, et al. (2006) Keap1 Recruits Neh2 through Binding to ETGE and DLG Motifs: Characterization of the Two-Site Molecular Recognition Model. Mol Cell Biol 26: 2887-2900.

7. Pi J, Qu W, Reece JM, Kumagai Y, Waalkes MP (2003) Transcription factor Nrf2 activation by inorganic arsenic in cultured keratinocytes: involvement of hydrogen peroxide. Exp Cell Res 290: 234-245.
8. Jisa E, Jungbauer A (2003) Kinetic analysis of estrogen receptor homo- and heterodimerization in vitro. J Steroid Biochem Mol Biol 84: 141-148.
9. Stenoien DL, Nye AC, Mancini MG, Patel K, Dutertre M, et al. (2001) Ligand-mediated assembly and real-time cellular dynamics of estrogen receptor alpha-coactivator complexes in living cells. Mol Cell Biol 21: 4404-4412.
10. Becker M, Baumann C, John S, Walker DA, Vigneron M, et al. (2002) Dynamic behavior of transcription factors on a natural promoter in living cells. EMBO Rep 3: 1188-1194.
11. McNally JG, Muller WG, Walker D, Wolford R, Hager GL (2000) The glucocorticoid receptor: rapid exchange with regulatory sites in living cells. Science 287: 1262-1265.
12. Kwak MK, Itoh K, Yamamoto M, Kensler TW (2002) Enhanced expression of the transcription factor Nrf2 by cancer chemopreventive agents: role of antioxidant response element-like sequences in the nrf2 promoter. Mol Cell Biol 22: 2883-2892.
13. Nguyen T, Sherratt PJ, Nioi P, Yang CS, Pickett CB (2005) Nrf2 controls constitutive and inducible expression of ARE-driven genes through a dynamic pathway involving nucleocytoplasmic shuttling by Keap1. J Biol Chem 280: 32485-32492.
14. Arava Y, Wang Y, Storey JD, Liu CL, Brown PO, et al. (2003) Genome-wide analysis of mRNA translation profiles in Saccharomyces cerevisiae. Proc Natl Acad Sci U S A 100: 3889-3894.
15. Jackson DA, Pombo A, Iborra F (2000) The balance sheet for transcription: an analysis of nuclear RNA metabolism in mammalian cells. Faseb J 14: 242-254.
16. Chan K, Kan YW (1999) Nrf2 is essential for protection against acute pulmonary injury in mice. Proc Natl Acad Sci U S A 96: 12731-12736.
17. Chan JY, Kwong M (2000) Impaired expression of glutathione synthetic enzyme genes in mice with targeted deletion of the Nrf2 basic-leucine zipper protein. Biochim Biophys Acta 1517: 19-26.
18. Leung L, Kwong M, Hou S, Lee C, Chan JY (2003) Deficiency of the Nrf1 and Nrf2 transcription factors results in early embryonic lethality and severe oxidative stress. J Biol Chem 278: 48021-48029.
19. Hayashi A, Suzuki H, Itoh K, Yamamoto M, Sugiyama Y (2003) Transcription factor Nrf2 is required for the constitutive and inducible expression of multidrug resistance-associated protein 1 in mouse embryo fibroblasts. Biochem Biophys Res Commun 310: 824-829.
20. Zhu H, Itoh K, Yamamoto M, Zweier JL, Li Y (2005) Role of Nrf2 signaling in regulation of antioxidants and phase 2 enzymes in cardiac fibroblasts: protection against reactive oxygen and nitrogen species-induced cell injury. FEBS Lett 579: 3029-3036.
21. Ishii T, Itoh K, Takahashi S, Sato H, Yanagawa T, et al. (2000) Transcription factor Nrf2 coordinately regulates a group of oxidative stress-inducible genes in macrophages. J Biol Chem 275: 16023-16029.
22. Sekhar KR, Long M, Long J, Xu ZQ, Summar ML, et al. (1997) Alteration of transcriptional and post-transcriptional expression of gamma-glutamylcysteine synthetase by diethyl maleate. Radiat Res 147: 592-597.
23. Liu RM, Gao L, Choi J, Forman HJ (1998) gamma-glutamylcysteine synthetase: mRNA stabilization and independent subunit transcription by 4-hydroxy-2-nonenal. Am J Physiol 275: L861-869.
24. Chen Y, Shertzer HG, Schneider SN, Nebert DW, Dalton TP (2005) Glutamate cysteine ligase catalysis: Dependence on ATPand modifier subunit for regulation of tissue glutathione levels. J Biol Chem.
25. Dalton TP, Dieter MZ, Yang Y, Shertzer HG, Nebert DW (2000) Knockout of the mouse glutamate cysteine ligase catalytic subunit (Gclc) gene: embryonic lethal when homozygous, and proposed model for moderate glutathione deficiency when heterozygous. Biochem Biophys Res Commun 279: 324-329.
26. Yang Y, Dieter MZ, Chen Y, Shertzer HG, Nebert DW, et al. (2002) Initial characterization of the glutamate-cysteine ligase modifier subunit Gclm(-/-) knockout mouse. Novel model system for a severely compromised oxidative stress response. J Biol Chem 277: 49446-49452.
27. Cho HY, Jedlicka AE, Reddy SP, Kensler TW, Yamamoto M, et al. (2002) Role of NRF2 in protection against hyperoxic lung injury in mice. Am J Respir Cell Mol Biol 26: 175-182.
28. Shen H, Ranganathan S, Kuzmich S, Tew KD (1995) Influence of ethacrynic acid on glutathione S-transferase pi transcript and protein half-lives in human colon cancer cells. Biochem Pharmacol 50: 1233-1238.

29. Jones BR, Li W, Cao J, Hoffman TA, Gerk PM, et al. (2005) The role of protein synthesis and degradation in the post-transcriptional regulation of rat multidrug resistance-associated protein 2 (Mrp2, Abcc2). Mol Pharmacol 68: 701-710.
30. Njalsson R, Norgren S, Larsson A, Huang CS, Anderson ME, et al. (2001) Cooperative binding of gamma-glutamyl substrate to human glutathione synthetase. Biochem Biophys Res Commun 289: 80-84.
31. Dinescu A, Cundari TR, Bhansali VS, Luo JL, Anderson ME (2004) Function of conserved residues of human glutathione synthetase: implications for the ATP-grasp enzymes. J Biol Chem 279: 22412-22421.
32. Benlloch M, Ortega A, Ferrer P, Segarra R, Obrador E, et al. (2005) Acceleration of glutathione efflux and inhibition of gamma-glutamyltranspeptidase sensitize metastatic B16 melanoma cells to endothelium-induced cytotoxicity. J Biol Chem 280: 6950-6959.
33. Dethmers JK, Meister A (1981) Glutathione export by human lymphoid cells: depletion of glutathione by inhibition of its synthesis decreases export and increases sensitivity to irradiation. Proc Natl Acad Sci U S A 78: 7492-7496.
34. Meister A, Anderson ME (1983) Glutathione. Annu Rev Biochem 52: 711-760.
35. Gilbert HF (1990) Molecular and cellular aspects of thiol-disulfide exchange. Adv Enzymol Relat Areas Mol Biol 63: 69-172.
36. Salerno M, Loechariyakul P, Saengkhae C, Garnier-Suillerot A (2004) Relation between the ability of some compounds to modulate the MRP1-mediated efflux of glutathione and to inhibit the MRPl-mediated efflux of daunorubicin. Biochem Pharmacol 68: 2159-2165.
37. Tang SS, Chang GG (1995) Steady-state kinetics and chemical mechanism of octopus hepatopancreatic glutathione transferase. Biochem J 309 (Pt 1): 347-353.
38. Ishikawa T, Esterbauer H, Sies H (1986) Role of cardiac glutathione transferase and of the glutathione S-conjugate export system in biotransformation of 4-hydroxynonenal in the heart. J Biol Chem 261: 1576-1581.
39. Widersten M, Kolm RH, Bjornestedt R, Mannervik B (1992) Contribution of five amino acid residues in the glutathione-binding site to the function of human glutathione transferase P1-1. Biochem J 285 (Pt 2): 377-381.
40. Ninomiya M, Ito K, Hiramatsu R, Horie T (2006) Functional analysis of Mouse and Monkey Multidrug resistance-associated protein 2 (Mrp2). Drug Metab Dispos.
