# Supplementary material for: Dose Response Relationship in Anti-Stress Gene Regulatory Networks
Source: PLoS Comput Biol. 2007 Mar 2;3(3):e24. doi: 10.1371/journal.pcbi.0030024 (PMC1808489; doi:10.1371/journal.pcbi.0030024)
Supplement: Supplementary file 14 [file pcbi.0030024.st005.pdf]

Table S5. Species, ODEs, and initial values for the anti-electrophilic gene regulatory network model.

Species	ODE	Initial concentration (nM)	Note
ATP	0	5×10^6	Adenosine triphosphate. The reported physiological concentration ranges between 1~7 mM [1-4].
Glu	0	10×10^6	Glutamate. The reported physiological concentration ranges between 4~30 mM [5-8].
Cys	0	0.3×10^6	Cysteine. Since GCL-catalyzed GSH synthesis is sensitive to cysteine level, and the K_m of cysteine in this reaction is 0.22 mM [9], we set the basal concentration at 0.3 mM so that cysteine is not completely saturating the reaction.
γ-GC	$f_{54}-f_{55}$	0.15×10^6	gamma-glutamylcysteine. In human fibroblast, the level of γ-GC was estimated to be 0.15 mM, given [GSH] is at 5mM [10].
Gly	0	1×10^6	Glycine. The concentration was assumed.
GSH	$f_{55}-f_{56}-f_{57}$	5×10^6	Reduced glutathione. The reported intracellular GSH concentration ranges from 1 to 10 mM in various biological tissue and cells [11]. We used 5 mM.
X	$f_{59}+f_{60}-f_{57}$	1×10^3	Here X represent an active electrophile. A major endogenous electrophile is the lipid peroxidation product 4-hydroxnonenal, the concentration of which ranges from 0.1~3 uM [12,13]. We used 1 uM.
GSX	$f_{57}-f_{58}$	200	GSH conjugate of X. This basal concentration is set at about 25% of the Ki for inhibition of GST.
Keap1	$f_1-f_3-f_5-f_6$	20	Kelch-like ECH-associated protein 1. The intracellular concentration is unknown. The value here gives the desired control on Nrf2 half-live as specified in Table S4.
$Keap1_o$	$f_2-f_4+f_5$	0	Oxidized or conjugated form of Keap1.
Nrf2	$-f_3-f_4-f_7/V_c$ $+f14-f15$	0.5	Nuclear factor erythroid 2 related factor 2 in the cytosol. The intracellular concentration is unknown. Since transcription factors generally exist in low abundance (in the nanomolar range), we implemented a basal level of 0.5 nM.
Nrf2-Keap1	$-f_1+f_3-f_6$	0	Complex of Nrf2 and Keap1.
Nrf2-$Keap1_o$	$-f_2+f_4+f_6$	0	Complex of Nrf2 and $Keap1_o$.
$Nrf2_n$	$f_7/V_n-f_8-f_9$	1	Nuclear Nrf2. As it appears that nuclear Nrf2 level is about twice that in the cytosol [14], we implemented a basal level of 1 nM.
Maf	$-f_9$	1	Small maf protein. The intracellular concentration is unknown. A low concentration of 1 nM was assumed since it is a transcription factor partner.
Nrf2-Maf	$f_9-f_{10}-3f_{16}-3f_{22}$ $-2f_{30}-2f_{38}-2f_{46}$	0	Heterodimer of Nrf2 and Maf.
$EpRE_{gene}$, where $gene\in\{Nrf2,Gclc,Gclm,Gs,Gst,Mrp\}$	$-f_m$, where $m\in\{10,16,22,30,38,46\}$	0.0167	EpRE-containing promoter for *Nrf2*, *Gclc*, *Gclm*, *Gs*, *Gst*, and *Mrp* genes, respectively. The value of 0.0167 is equivalent to one copy of molecule in a nuclear volume of 100 um^3.

Nrf2-Maf-EpRE$_{gene}$, where $gene \in \{Nrf2, Gclc, Gclm, Gs, Gst, Mrp\}$	f_m, where $m \in \{10,16,22,30,38,46\}$	0	Complex of Nrf2-Maf dimer and EpRE-containing promoter for *Nrf2*, *Gclc*, *Gclm*, *Gs*, *Gst*, and *Mrp* genes, respectively.
Gene$_{off}$, where $gene \in \{Nrf2, Gclc, Gclm, Gs, Gst, Mrp\}$	$-f_n$, where $n \in \{11,17,23,31,39,47\}$	0.0167	Inactive state of *Nrf2*, *Gclc*, *Gclm*, *Gs*, *Gst*, and *Mrp* genes, respectively. The value of 0.0167 is equivalent to one copy of gen in a nuclear volume of 100 um^3.
Gene$_{on}$, where $gene \in \{Nrf2, Gclc, Gclm, Gs, Gst, Mrp\}$	f_n, where $n \in \{11,17,23,31,39,47\}$	0	Active state of *Nrf2*, *Gclc*, *Gclm*, *Gs*, *Gst*, and *Mrp* genes, respectively.
Gene mRNA, where $Gene \in \{Nrf2, Gclc, Gclm, Gs, Gst, Mrp\}$	$f_p V_n / V_c - f_q$, where $p \in \{12,18,24,32,40,48\}$ $q \in \{13,19,25,33,41,49\}$	0	mRNA of *Nrf2*, *Gclc*, *Gclm*, *Gs*, *Gst*, and *Mrp* genes, respectively.
GCLC	$f_{20} - f_{21} - f_{28}$	1.7×10^3	Glutamate cysteine ligase catalytic subunit. See the GCLM and GCL entries in the same table.
GCLM	$f_{26} - f_{27} - f_{28}$	0.36×10^3	Glutamate cysteine ligase modifier subunit. At this concentration, the GCLC/GCL ratio is 3 at the basal condition, as specified in reaction 28 in Table S4.
GCL	$f_{28} - f_{29}$	0.6×10^3	Glutamate cysteine ligase holoenzyme. This concentration, together with that of GCLC, gives a basal GSH synthesis rate of 386 nM/s, which is close to 360 nM/s measured in B16M melanoma cells given a cell volume of 1000 um^3 [15]
GS$_{mono}$	$f_{34} - f_{35} - 2f_{36}$	0	Glutathione synthetase monomer.
GST$_{mono}$	$f_{42} - f_{43} - 2f_{44}$	0	Glutathione S-transferase monomer.
MRP$_{mono}$	$f_{50} - f_{51} - 2f_{52}$	0	Multidrug resistance-associated protein monomer.
GS	$f_{36} - f_{37}$	0.96×10^3	Glutathione synthetase dimer. This concentration keeps γ-GC at 0.15 mM at the basal condition. See the γ-GC entry in the same table for the choice of 0.15 mM.
GST	$f_{44} - f_{45}$	0.185×10^3	Glutathione S-transferase dimer. This concentration keeps X at 1 uM at the basal condition. See the X entry in the same table for the choice of 1 uM.
MRP	$f_{52} - f_{53}$	7.22×10^3	Multidrug resistance-associated protein. This concentration keeps GSX at 0.2 uM at the basal condition.

References

1. Ko SH, Lee SK, Han YJ, Choe H, Kwak YG, et al. (1997) Blockade of myocardial ATP-sensitive potassium channels by ketamine. Anesthesiology 87: 68-74.
2. Gribble FM, Loussouarn G, Tucker SJ, Zhao C, Nichols CG, et al. (2000) A novel method for measurement of submembrane ATP concentration. J Biol Chem 275: 30046-30049.
3. Leist M, Single B, Castoldi AF, Kuhnle S, Nicotera P (1997) Intracellular adenosine triphosphate (ATP) concentration: a switch in the decision between apoptosis and necrosis. J Exp Med 185: 1481-1486.
4. Marcussen M, Larsen PJ (1996) Cell cycle-dependent regulation of cellular ATP concentration, and depolymerization of the interphase microtubular network induced by elevated cellular ATP concentration in whole fibroblasts. Cell Motil Cytoskeleton 35: 94-99.
5. Goldstein L (1966) Relation of glutamate to ammonia production in the rat kidney. Am J Physiol 210: 661-666.

6. Geerts WJ, Jonker A, Boon L, Meijer AJ, Charles R, et al. (1997) In situ measurement of glutamate concentrations in the periportal, intermediate, and pericentral zones of rat liver. J Histochem Cytochem 45: 1217-1229.
7. Divino Filho JC, Hazel SJ, Furst P, Bergstrom J, Hall K (1998) Glutamate concentration in plasma, erythrocyte and muscle in relation to plasma levels of insulin-like growth factor (IGF)-I, IGF binding protein-1 and insulin in patients on haemodialysis. J Endocrinol 156: 519-527.
8. Huang CS, Chang LS, Anderson ME, Meister A (1993) Catalytic and regulatory properties of the heavy subunit of rat kidney gamma-glutamylcysteine synthetase. J Biol Chem 268: 19675-19680.
9. Chen Y, Shertzer HG, Schneider SN, Nebert DW, Dalton TP (2005) Glutamate cysteine ligase catalysis: Dependence on ATPand modifier subunit for regulation of tissue glutathione levels. J Biol Chem.
10. Ristoff E, Hebert C, Njalsson R, Norgren S, Rooyackers O, et al. (2002) Glutathione synthetase deficiency: is gamma-glutamylcysteine accumulation a way to cope with oxidative stress in cells with insufficient levels of glutathione? J Inherit Metab Dis 25: 577-584.
11. Meister A, Anderson ME (1983) Glutathione. Annu Rev Biochem 52: 711-760.
12. Esterbauer H, Zollner H (1989) Methods for determination of aldehydic lipid peroxidation products. Free Radic Biol Med 7: 197-203.
13. Esterbauer H, Eckl P, Ortner A (1990) Possible mutagens derived from lipids and lipid precursors. Mutat Res 238: 223-233.
14. Pi J, Qu W, Reece JM, Kumagai Y, Waalkes MP (2003) Transcription factor Nrf2 activation by inorganic arsenic in cultured keratinocytes: involvement of hydrogen peroxide. Exp Cell Res 290: 234-245.
15. Benlloch M, Ortega A, Ferrer P, Segarra R, Obrador E, et al. (2005) Acceleration of glutathione efflux and inhibition of gamma-glutamyltranspeptidase sensitize metastatic B16 melanoma cells to endothelium-induced cytotoxicity. J Biol Chem 280: 6950-6959.
